# Nonlinear nanophotonics for high-dimensional quantum states

**DOI:** 10.1038/s41377-025-02179-0

**Published:** 2026-01-29

**Authors:** Liat Nemirovsky-Levy, Amit Kam, Meir Lederman, Meir Orenstein, Uzi Pereg, Guy Bartal, Mordechai Segev

**Affiliations:** 1 Physics department, Technion – Israel Institute of Technology, Haifa, Israel; 2 Solid state institute, Technion – Israel Institute of Technology, Haifa, Israel; 3 Andrew and Erna Viterbi Department of Electrical & Computer Engineering, Technion – Israel Institute of Technology, Haifa, Israel

**Keywords:** Quantum optics, Nanophotonics and plasmonics

## Abstract

Quantum nanophotonics merges the precision of nanoscale light manipulation with the capabilities of quantum technologies, offering a pathway for enhanced light-matter interaction and compact realization of quantum devices. Here, we show how a recently-demonstrated nonlinear nanophotonic process can be employed to selectively create photonic high-dimensional quantum states (qudits). We utilize the nonlinearity on the surface of the nanophotonic device to dress, through the polarization of the pump field, the near-field modes carrying angular momentum and their superpositions. This idea is an important step towards experimental realizations of quantum state generation and manipulation through nonlinearity within nanophotonic platforms, and enables new capabilities for on-chip quantum devices.

## Introduction

In recent years, there has been growing interest in developing quantum optics at the nanoscale, by incorporating new capabilities of photonic circuitry into nanophotonic platforms^1–5^. Quantum optics at the nanoscale holds great promise for advancing future quantum information applications, such as transmission^6^, security, distributed computation^7,8^ etc., as well as a promising platform for quantum computing^9,10^. Its compact size and compatibility with current on-chip technologies makes it particularly appealing^11,12^. Additionally, the tight confinement of the light in nanophotonic platforms enhances nonlinear effects such as spontaneous parametric down-conversion (SPDC)^13–15^ and four-wave-mixing^16^, giving rise to high flux of entangled photons. The combination of nanophotonics, nonlinear optics and quantum optics offers greater ability to control, generate, and manipulate quantum states of light. Moreover, the tight confinement of light in nanophotonics, manifested in surface modes whose intensity peaks at the surface and enable enhanced light-matter interactions, providing unique opportunities for quantum state control.

However, generating, controlling and manipulating quantum states at the nanoscale also presents new challenges, particularly in applying external control knobs at the sub-wavelength scale. Nonlinear optics can offer a powerful approach for mitigating these challenges by providing additional interaction mechanisms^17,18^, which enable precise manipulation of quantum states and their interactions. These nonlinear processes have been successfully employed in various macroscopic quantum technologies, including entanglement generation, quantum frequency conversion^19,20^, generating squeezed states of light^21,22^, etc. While the integration of such nonlinear techniques into a nanophotonic platform is not straightforward, recent advances^23^ suggest that judicious design of nanostructures can harness these processes effectively, unlocking their full potential at the nanoscale.

Nanophotonic platforms enable strong field confinement, enhancing nonlinear parametric processes, and thus leads to higher generation rates of the desired photon states. Moreover, while nanophotonic platforms enable strong field confinement, thereby enhancing nonlinear parametric processes, the key challenge is to translate these advantages into higher entangled photon generation rates and into methods for efficiently coupling specific quantum states into free space for measurement and application. A recent advance, developed in our lab^24^, can directly address this selective extraction of desired states from the of near-field by harnessing the optical nonlinearity inherent to metals which often comprise nanophotonic systems.

Here, we propose a new concept for generation and manipulation of high dimensional quantum states in a nanophotonic platform, exploiting its optical nonlinearity. We present a method to convert near-field modes carrying angular momentum into far-field photonic states encoded in polarization and orbital angular momentum (OAM) degrees of freedom. This is achieved through a nonlinear interaction that projects the near-field states onto specific spin and OAM states in the far field photons, enabling direct readout of the quantum information initially encoded in the near-field. This nanophotonic setting can be used as a highly compact system for generating and control in qudits. These platforms enable robust integration with on-chip photonic technologies, enhanced scalability, and resilience to environmental perturbations, making it uniquely suited for practical, miniaturized quantum communication devices.

A central advantage of such a platform is its ability to realize high-dimensional quantum states, or qudits - quantum states with more than two levels – which represent a promising avenue for quantum information processing^25^. They allow encoding and processing of more information per mode compared to qubits^26–30^, utilize entanglement more efficiently, and enable more compact systems. This is particularly relevant for quantum communications and teleportation of high-dimensional states^31–33^, as well as for implementing high-dimensional quantum error-correcting codes^34,35^. Techniques to generate such high-dimensional states have so far relied on bulk optical setups with spatial light modulators^36^, time-bin encoding in fiber systems^37–40^, or integrated photonic platforms utilizing waveguide arrays and microring resonators^41–43^. While these approaches are effective, they often require complex interferometric stabilization or large-scale integration. Nanophotonic platforms, with enhanced nonlinear interactions and control over multiple photonic modes - such as angular momentum states^44^ or frequency-comb-encoded states^45^, can offer a route toward compact, high-density, scalable generation of qudits.

### Angular momentum in nanophotonic systems

The total angular momentum (TAM) of light is a fundamentally conserved quantity, both classically and quantum mechanically. In specific settings such as paraxial optics, the angular momentum of the photons can be uniquely divided into spin angular momentum (SAM) and orbital angular momentum (OAM). In nanophotonic settings, which impose confinement to the sub-wavelength scale, SAM and OAM cannot be trivially separated. However, in the near-field regime, while SAM and OAM remain inseparable, photons can couple to modes characterized by their TAM, that serves as a good quantum number^46–48^. Such near-field modes carrying TAM can be entangled^44^.

Traditionally, imaging and detection of near-field modes has posed a problem. This was recently resolved in a technique facilitating real-time imaging of near-field modes, relying on nonlinear polarization-dependent coupling of the nanophotonic modes to far-field modes via four-wave-mixing^24^. In that scheme, the polarization of the pump field determines the polarization of the emitted photons in the far-field modes. The far-field modes can be engineered to be paraxial, such that the SAM and OAM are separable, facilitating complete reconstruction of the quantum properties originally encoded on the photons in the near-field modes. In this nonlinear imaging process, the TAM of the emitted light matches that of the nanophotonic mode. Consequently, because the TAM of the photons is the sum of the SAM and OAM, and the SAM of the measured far-field photons matches the SAM of the pump field, the measurement process enables selective measurement of the OAM of the photons, and full recovery of the TAM of the photons in the near-field modes of the nanophotonic system.

In this work, we extend this concept to the quantum regime. We consider nanophotonic system supporting multiple modes carrying different TAM per photon, and exploit nonlinear interactions to encode and decode quantum information onto multi-level photonic states. Thus, the nonlinear interaction in the near field can generate high-dimensional quantum states carried by the photons that are coupled out from the nanophotonic platform. These high dimensional quantum states can be controlled, at will, by the pump beam.

In the following section, we explain how the angular momentum of the emitted photons can be controlled using the nonlinear interaction.

## Results

### Designing the generation of high-dimensional quantum states in a nanophotonic platform

**Fig. 1:**
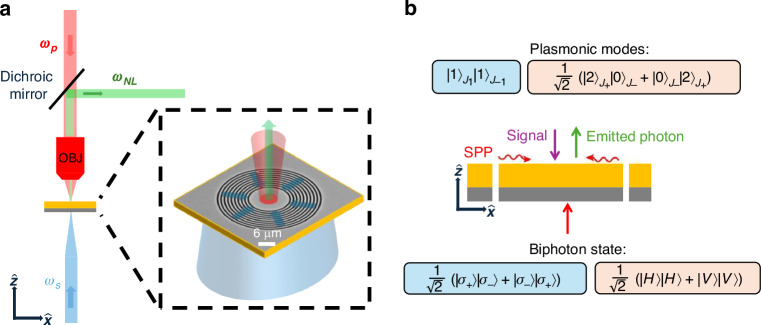
**a** Proposed setup for quantum nonlinear near-field generation of the photonic states. A photonic pump beam (red) is focused onto the sample. The plasmonic field is generated by coupling single photons (blue) into the nanophotonic modes of the sample. The nonlinear interaction yields a single photon, marked by the green arrow, coupled to far-field modes. This set-up is taken from the classical experiment in ref. ^24^. **b** Schematic cross-section of the nanophotonic sample. The straight red arrow represents the incident biphoton state, while the wavy red arrow denotes the surface plasmons. The purple arrow indicates the signal beam, and the green arrow marks the emitted photon from the four-wave mixing process. The SPP modes generated depend on the polarization of the classical signal, which dictates the polarization state of the biphotons: pink corresponds to linear basis polarization, and blue to circular basis polarization

We first describe the generation of high-dimensional quantum states in our nanophotonic system, as shown in Fig. 1a. The setup consists of a thick gold layer supporting surface plasmon polaritons (SPPs) at the gold-air interface, with a circular coupler that transforms the SAM of an input photon into a transverse magnetic (TM) Bessel-like plasmonic mode characterized by its TAM, whose value is equal to the value of the original SAM of the impinging photon.

We treat the surface plasmon polariton (SPP) supported by the gold slab as a quantized electromagnetic mode (Fig. 1b). In general, one expands the electric field operator in terms of mode functions and bosonic ladder operators. For a single SPP mode of frequency ${\omega }_{{SPP}}$, one can write:

$${\hat{E}}_{{spp}}\left({\boldsymbol{r}},t\right)={\hat{E}}_{{spp}}^{(+)}\left({\boldsymbol{r}},t\right)+{\hat{E}}_{{spp}}^{(-)}\left({\boldsymbol{r}},t\right)$$

With ${\hat{E}}_{{spp}}^{(+)}\left({\boldsymbol{r}},t\right)={E}_{{SPP}}\left(r\right){\hat{a}}_{{SPP}}{e}^{-i{\omega }_{{SPP}}t}$, ${\hat{E}}_{{spp}}^{(-)}={\left({\hat{E}}_{{spp}}^{(+)}\right)}^{\dagger }$. Here, ${\hat{a}}_{{SPP}}$ is the annihilation operator for the SPP mode (and ${\hat{a}}_{{SPP}}^{\dagger }$ its creation operator).

The complex vector ${E}_{{spp}}({\boldsymbol{r}})$ is the spatial mode profile of the SPP field. The normalization is chosen such that: ${\varepsilon }_{0}\int {d}^{3}r{\left|{E}_{{SPP}}\left(r\right)\right|}^{2}=\frac{\hslash {\omega }_{{SPP}}}{2}$. (This ensures that one quantum excitation in the SPP carries energy $\hslash {\omega }_{{SPP}}$. In cylindrical coordinates $\left(r,\phi ,z\right)$, the mode profile can be expressed as:

1 $${E}_{{SPP}}\left(r\right)\propto {E}_{0,{SPP}}\left[\left(-\hat{r}+i\hat{\phi }\right){J}_{n-1}\left({k}_{r}r\right){e}^{i\left(n-1\right)\phi }+\left(\hat{r}+i\hat{\phi }\right){J}_{n+1}\left({k}_{r}r\right){e}^{i\left(n+1\right)\phi }\right]+{J}_{n}\left({k}_{r}r\right){e}^{{in}\phi }\hat{z}$$

Where $n$ is an integer denoting the angular momentum quantum number. Each component carries OAM $L$ and spin $\sigma$ such that the TAM $J=L+\sigma =n\hslash$.

The third-order nonlinearity ${\chi }^{(3)}$ of gold enables the nonlinear interaction between the classical pump, the quantized SPP mode, and the quantized emitted photon. The nonlinear polarization is: ${P}^{(3)}={\varepsilon }_{0}{\chi }^{(3)}{{\boldsymbol{E}}}^{3}$. The corresponding interaction Hamiltonian is: ${\hat{H}}_{I}=-\frac{{\varepsilon }_{0}}{4}\int {d}^{3}r{\chi }_{{ijkl}}^{\left(3\right)}{\hat{E}}^{i}{\hat{E}}^{j}{\hat{E}}^{k}{\hat{E}}^{l}$. We decompose the total field as: $\hat{E}={E}_{{pump}}+{\hat{E}}_{{SPP}}+{\hat{E}}_{{out}}$. Keeping the term corresponding to two pump photons, one SPP photon, and one emitted photon, the interaction Hamiltonian becomes:

2 $${\hat{H}}_{I}\approx -{\varepsilon }_{0}{\chi }^{\left(3\right)}{\int {d}^{3}r\left[{E}_{{pump}}\left({\boldsymbol{r}},t\right)\right]}^{2}{\hat{E}}_{{spp}}^{\left(+\right)}\left({\boldsymbol{r}},t\right)\cdot {\hat{E}}_{{out}}^{\left(-\right)}\left({\boldsymbol{r}},t\right)+h.c$$

Where ${E}_{{pump}}\left({\boldsymbol{r}},t\right)={E}_{0,{pump}}\left({\boldsymbol{r}}\right){e}^{-i{\omega }_{p}t}+c.c$, ${\hat{E}}_{{spp}}^{\left(+\right)}\left({\boldsymbol{r}},t\right)={E}_{{SPP}}\left({\boldsymbol{r}}\right){\hat{a}}_{{SPP}}{e}^{-i{\omega }_{{SPP}}t},\,{\hat{E}}_{{out}}^{\left(-\right)}\left({\boldsymbol{r}},t\right)={E}_{{out}}^{* }\left(r\right){\hat{a}}_{{out}}^{\dagger }{e}^{i{\omega }_{{out}}t}$ Under energy conservation $2{\omega }_{p}={\omega }_{{SPP}}+{\omega }_{{out}}$, we get:

3 $${\hat{H}}_{I}=\hslash g{\hat{a}}_{{out}}^{\dagger }{\hat{a}}_{{SPP}}+\hslash {g}^{* }{\hat{a}}_{{SPP}}^{\dagger }{\hat{a}}_{{out}}$$

With coupling constant: $g=-\frac{{\varepsilon }_{0}{{\rm{\chi }}}^{\left(3\right)}}{\hslash }\int {d}^{3}r{E}_{0,{pump}}^{2}\left(r\right){u}_{{SPP}}\left(r\right){u}_{{out}}^{* }\left(r\right)$.

In the case where the pump is circularly polarized: ${E}_{{pump}}\left({\boldsymbol{r}},t\right)={E}_{0,{pump}}\left({\boldsymbol{r}}\right){\hat{{\boldsymbol{\sigma }}}}_{\pm }{e}^{-i{\omega }_{p}t}+c.c$, with ${\hat{\boldsymbol{\sigma}}}_{\pm}=\frac{1}{\sqrt{2}}\left(\hat{x} + i \hat{y}\right)$, the nonlinear polarization points in the same direction. The emitted field operator becomes:

4 $${\hat{E}}_{{out}}\left({\boldsymbol{r}},t\right)\propto {{\rm{\chi }}}^{\left(3\right)}{E}_{0,{pump}}^{2}\left({\boldsymbol{r}}\right)\left[{E}_{{SPP}}^{* }\left({\boldsymbol{r}}\right){\hat{{\boldsymbol{\sigma }}}}_{\pm }\right]{\hat{a}}_{{out}}^{\dagger }{{e}^{-i{\omega }_{{out}}t}\hat{{\boldsymbol{\sigma }}}}_{\pm }$$

Each output polarization component is proportional to the conjugate of the SPP field component projected onto the pump polarization. Substituting the SPP mode (Eq. (1)) yields:

5 $${\hat{E}}_{{out}}\left({\boldsymbol{r}},t\right)\propto {E}_{0,{pump}}^{2}{E}_{0,{SPP}}\left(\begin{array}{c}-{k}_{z}{k}_{r}{J}_{n-1}\left({k}_{r}r\right){e}^{i\left(n-1\right)\phi }\\ {k}_{z}{k}_{r}{J}_{n+1}\left({k}_{r}r\right){e}^{i\left(n+1\right)\phi }\\ {{k}_{r}^{2}J}_{n}\left({k}_{r}r\right){e}^{{in}\phi }\end{array}\right){\hat{a}}_{{out}}^{\dagger }\,{e}^{i{k}_{z}\cdot z-i{\omega }_{{out}}t}$$

This result clearly shows that the output field components are determined by the in-plane rotating field structure of the surface plasmon polariton (SPP) mode, specifically through the projection of the complex-conjugated SPP field ${{\hat{E}}^{\star }}_{{SPP}}\left(r\right)$ onto the circular polarization of the classical pump, ${\hat{\sigma }}_{\pm }$. That is, the emitted electric field operator satisfies Eq. (4), indicating that the output photon inherits the pump’s circular polarization and angular momentum. Substituting the explicit spatial form of the SPP mode, one finds that the emitted photon carries well-defined OAM components of $\left(n-1\right)\hslash ,\,\left(n+1\right)\hslash$, and $n\hslash$ in the ${\sigma }_{+}$, ${\sigma }_{-}$ and $z$ polarization directions, respectively, with a total angular momentum $J=n\hslash$ preserved in each component. Consequently, the nonlinear interaction facilitates the emission of a single photon whose vectorial and angular momentum characteristics are fully determined by the pump polarization and the underlying near-field structure. This process enables deterministic control over the emitted photon’s polarization and OAM content via the handedness of the classical pump field.

Next, we consider the coupling of a biphoton state into the nanophotonic system, allowing to explore the nonlinear interaction with near-field modes. This process begins by generating an entangled photon pair from a type-II spontaneous parametric down-conversion (SPDC) crystal, which in the circular polarization basis, is described as:

6 $${\left|\psi \right\rangle }_{AB}=\tfrac{1}{\sqrt{2}}\left({\left|{\sigma }_{+}\right\rangle }_{A}{\left|{\sigma }_{-}\right\rangle }_{B}+{\left|{\sigma }_{-}\right\rangle }_{A}{\left|{\sigma }_{+}\right\rangle }_{B}\right)$$

Here, |σ_+_〉, |σ_-_〉 indicate right and left circular polarizations, respectively. Labels $A$ and $B$ refer to the two photons. As explained above, the polarization of the output photon is determined by the polarization of the pump in the four-wave-mixing process.

To obtain the plasmonic mode states generated by the coupled biphoton, we make use of the vectorial structure of the surface modes. Specifically, a near-field mode with TAM $J=n\hslash$ can be represented as a superposition of circularly polarized in-plane components: the ${\sigma }_{-}$ component associated with a $\left(n+1\right)$-order Bessel distribution (OAM $L=n+1$), the ${\sigma }_{+}$ component with a $\left(n-1\right)$-order Bessel distribution (OAM $L=n-1$), and a longitudinal component with OAM $L=n$. In all cases the relation $J=L+\sigma$ holds, ensuring consistency of the angular momentum decomposition.

Thus, coupling the biphoton state from Eq. (6), the following nanophotonic plasmonic modes achieved on the surface:

7 $${\left|\psi\right\rangle}_{AB}\;\to\;{\left|1_{J_1}\right\rangle}_{A}\,{\left|1_{J_{-1}}\right\rangle}_{B}$$

Where $\left|{\rm{\psi }}\right\rangle$ is the initial biphoton state coupled into the nanophotonic system, $\left|{\rm{n,m }}\right\rangle$ are Fock states corresponding to the rotating plasmonic mode basis which consists of Bessel modes with TAM values $+\hslash$ and $-\hslash$ for ${J}_{1},{J}_{-1}$ respectively. For the derivation of the nanophotonic states, see the supplementary information.

Up to this point, we have two distinct levels stored in the nanophotonic quantum states encoded by their TAM values. Now, to generate the final qudit state, we extract the photons from the system to a measurement. This is achieved through the nonlinear parametric process between the SPP modes and an intense classical field (“pump”), as described above. The two extracted photons, now propagating to the far field, are in different orthogonal entangled states of SAM and OAM values out of two possibilities:

8 $$\begin{array}{c}{\rm for}\quad \sigma_{+}\quad {\rm polarized\ pump}\to \,{\left|{\sigma }_{+}\right\rangle }_{{S}_{A}}\otimes \left({\left|{j}_{0}\right\rangle }_{{\rm{L}}}+{\left|{j}_{-2}\right\rangle }_{{\rm{L}}}\right)\\ {\rm for}\quad \sigma_{-}\quad {\rm polarized\ pump}\to \,{\left|{\sigma }_{-}\right\rangle }_{{S}_{A}}\otimes \left({\left|{j}_{0}\right\rangle }_{{\rm{L}}}+{\left|{j}_{2}\right\rangle }_{{\rm{L}}}\right)\end{array}$$

**Fig. 2:**
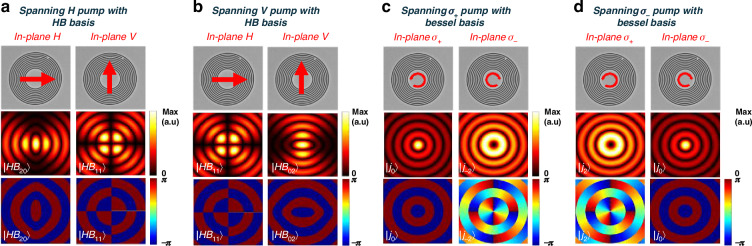
Spatial distributions of photonic states illustrating the amplitude and phase profiles based on the polarization of the pump. The spatial patterns are represented either by Bessel beams or Hermite-Bessel (HB) functions, which can be described as superpositions of Bessel beams. When the nonlinear process involves a circularly polarized pump, the emitted photons are described by Bessel beams (as shown in (**c**, **d**). In contrast, a linearly polarized pump (**a**, **b**) results in photon emission described by HB functions

Thus, the polarization of the emitted photons matches that of the pump field, while their TAM value corresponds to that of the interacting SPP mode. As a result, each of the extracted photons can now have one of four possible values (presented in Fig. 2c, d). For instance, if the pump field is circularly polarized, two values are retained according to the TAM of the plasmonic mode (${J}_{1}$ and ${J}_{-1}$) and two values corresponding to the two possibilities of polarization handedness of the pump field. Thus, given the four options of the emitted photon with degrees of freedom (DoFs) of its polarization and OAM, we can assign the digits 0,1,2,3 to the following four photonic states:

9 $$\left\{\begin{array}{l}{{\prime} {\prime}\atop }1^{{\prime} {\prime} }=\left|{\sigma }_{+},{j}_{0}\right\rangle \,\\ {{\prime} {\prime}\atop }2^{{\prime} {\prime} }=\left|{\sigma }_{-},\,{j}_{0}\right\rangle \,\\ \,{{\prime} {\prime}\atop}3^{{\prime} {\prime} }=\left|{\sigma }_{-},{j}_{2}\right\rangle \,\\ \,{{\prime} {\prime}\atop}0^{{\prime} {\prime} }=\left|{\sigma }_{+},{j}_{-2}\right\rangle \end{array}\right.$$

So far, we have analyzed the case where the pump field is circularly polarized. However, a similar process occurs for a linearly polarized pump field, where the zero TAM of the SPP modes (in the linear polarization basis) is preserved. This leads to a different set of four far-field quantum states, again forming a qudit basis. In this case, the biphoton input state coupled into the nanophotonic system is:

10 $${\left|\psi \right\rangle }_{{AB}}=\tfrac{1}{\sqrt{2}}\left({\left|H\right\rangle }_{A}{\left||V\right\rangle }_{B}+{\left|V\right\rangle }_{A}{\left||H\right\rangle }_{B}\right)$$

Where ${\left|{\rm{H}} \right\rangle },{\left|{\rm{V}} \right\rangle }$ indicate horizontal and vertical linear polarizations, respectively, and labels $A$ and $B$ refer to the two photons.

For convenience of tracking the nonlinear process we rewrite the dual mode nanophotonic state of Eq. 7 in the combination state:

11 $$\left|{\rm{\psi }}\right\rangle \to \tfrac{1}{\sqrt{2}}\left(\left|{2}_{{J}_{+}},{0}_{{J}_{-}}\right\rangle +\left|{0}_{{J}_{+}},{2}_{{J}_{-}}\right\rangle \right)$$

where, $\left|{\rm{n,m }}\right\rangle$ are Fock states corresponding to the plasmonic mode of azimuthal standing waves ${J}_{\pm }$ that are defined as ${J}_{\pm }=\frac{1}{\sqrt{2}}\left({J}_{1}\pm {J}_{-1}\right)$

When a linearly polarized pump field is applied, the far-field spatial profile of the extracted photons corresponds to Hermite-Bessel (HB) modes which are proper superpositions of the Bessel beams^49,50^. In this case, the two extracted photons in the far field, have the following characteristics:

The$\,\left|{2}_{{J}_{+}},{0}_{{J}_{-}}\right\rangle$ part in Eq. (11) yields a photon at a state ${\left|H\right\rangle }_{{S}_{A}}\otimes {\left|H{B}_{20}\right\rangle }_{L}$ for ${H}$ polarized pump, or ${\left|V\right\rangle }_{{S}_{A}}\otimes {\left|H{B}_{11}\right\rangle }_{L}$ for ${V}$ polarized pump. Similarly, the $\left|{0}_{{J}_{+}},{2}_{{J}_{-}}\right\rangle$ part results in ${\left|H\right\rangle }_{{{\rm{S}}}_{{\rm{A}}}}\otimes {\left|H{B}_{11}\right\rangle }_{L}$ for ${H}$ polarized pump, or ${\left|V\right\rangle }_{{S}_{A}}\otimes {\left|H{B}_{02}\right\rangle }_{{\rm{L}}}$ for ${V}$ polarized pump, where |*HB*〉_*nm*_ are Hermit-Bessel beams that do not carry angular momentum. For this configuration, we assign the four digits for the linearly polarized pump field as:

12 $$\left\{\begin{array}{l}{{\prime} {\prime}\atop }1^{{\prime} {\prime} }=\left|H,{{HB}}_{20}\right\rangle \\ {{\prime} {\prime} \atop}2^{{\prime} {\prime} }=\left|V,{{HB}}_{11}\right\rangle \\{{\prime} {\prime}\atop}3^{{\prime} {\prime} }=\left|H,{{HB}}_{11}\right\rangle \\ {{\prime} {\prime}\atop }0^{{\prime} {\prime} }=\left|V,{{HB}}_{02}\right\rangle \end{array}\right.$$

**Fig. 3:**
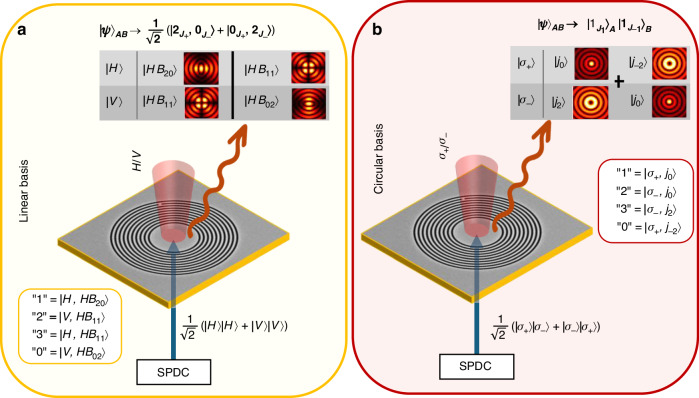
Qudit encoding in the far-field photon generated via the nonlinear interaction. **a** A linearly polarized pump and a biphoton state prepared in the linear polarization basis are coupled into the nanophotonic platform, resulting in a structured quantum plasmonic pattern. The nonlinear interaction leads to the emission of a single photon with four distinct possible outcomes, each corresponding to a different spatial or polarization mode, thereby encoding a four-level qudit. **b** Same as in (**a**), but using a circularly polarized pump and an input biphoton state prepared in the circular polarization basis. The emitted photon similarly encodes a qudit in four distinct circularly polarized vector modes

This approach allows for precise control over the generated qudit states, enabling flexible quantum information encoding. The two possibilities if the encoded qudits in the far field, depending on the polarization of the pump, can be seen in Fig. 3. The quantum state of the far-field photons can now be expressed on an extended basis as $\left|{S}_{A}\right\rangle \otimes \left|{\mathcal{l}}\right\rangle$, where ${S}_{A}$ represents its spin (polarization), and ${\mathcal{l}}$ is the OAM DoF. The polarization is a binary DoF, while the OAM DoF, also known as related to a topological charge ${\mathcal{l}}$, can take any value $\ell =m\hslash$, where $m$ is integer. The spin of the emitted photon is determined solely by the pump polarization, consequently the OAM of the emitted photon is determined by the subtraction of this spin from the TAM of the original near field mode. When the emitted photon is in a paraxial mode, the SAM and OAM of the far-field photon become separable, enabling to recover the quantum information encoded on the photon by projections onto SAM and OAM states separately.

By engineering multiple SPP modes with distinct azimuthal quantum numbers, this approach can, in principle, be extended to generate higher-dimensional polarization–OAM states, where each additional mode contributes two orthogonal basis states. However, scaling to larger dimensions remains experimentally challenging, as coupling and detection efficiencies for single photons decrease rapidly with the number of participating modes.

While quantitative values of the third-order susceptibility ${x}^{\left(3\right)}\sim 2\times {10}^{-18}{{\rm{m}}}^{2}/{{\rm{V}}}^{2}$ have been extracted from four-wave mixing experiments at gold interfaces using femtosecond pulses^24,51^, the regime of quantum-level nonlinear interactions - such as processes involving a single plasmon and a classical pump - has not yet been explored experimentally. Nonetheless, independent studies in quantum plasmonics have demonstrated that plasmons can couple to single or entangled photons while preserving quantum properties^44,52^. Taken together, these findings indicate that near-field enhancement in nonlinear processes and quantum plasmons can, in principle, coexist, which forms the basis for our theoretical investigation.

To highlight the feasibility of our approach, we propose the following structure which maximizes the nonlinear interaction strength using a structured metal–dielectric–semiconductor stack, specifically a gold–silica–silicon platform^53^. In this structure, gold provides the plasmonic localization, silica acts as a spacer and buffer layer, and the mode is primarily confined inside the silicon.

Using realistic parameters, we obtain a detected photon rate on the order of ${R}_{\det }\approx 118$ counts per second (see Supplementary Material section D for details). These values are well within the sensitivity range of superconducting nanowire single-photon detectors, indicating that in the gold–silica–silicon multilayer platform, photon-pair generation into the far field via nonlinear interactions is experimentally accessible.

Our goal is to connect nonlinear near-field processes with the encoding of quantum information in high-dimensional photonic degrees of freedom. Within this framework, we show that entangled input states can be transformed in a way that reflects the vectorial structure of the near-field plasmonic modes. This mechanism allows selective generation of quantum states with spatial and polarization structure determined by the classical pump configuration. The theoretical framework presented here offers a potential route for studying the interaction between nanophotonic field structure and quantum state control.

As an example for an application, detailed in the Supplementary Information (section B+C), we propose a quantum key distribution (QKD) protocol using the qudits encoded in the far-field photons. In this scheme, each digit of the key is associated with a choice of basis - linear or circular - for both the classical pump and the biphoton input. Building on the BB84 protocol^54,55^ our approach generalizes it to higher dimensions by employing qudits instead of qubits, thereby enabling an enhanced key rate. This aligns with previous studies that have also reported increased key rates in high-dimensional QKD^56,57^.

## Discussion

In summary, we presented a novel approach which employs nonlinear optics in nanophotonic platforms for the generation and manipulation of high-dimensional quantum states. By exploiting a nonlinear interaction and the properties of the total angular momentum carried by near field photons, our method enables the generation of photons with predesigned specific SAM and OAM combinations. The ability to control quantum states at the nanoscale paves the way for practical implementations of quantum information protocols, such as, for example, the multilevel QKD with enhanced key rates, analyzed in the supplementary information, due to the use of qudits instead of qubits, within compact, scalable, and robust nanophotonic devices. Notably, this work demonstrates how near-field properties can be utilized for encoding quantum information and executing communication tasks.

Looking forward, incorporating such nanophotonic platforms within on-chip technologies represents a significant step toward realizing scalable quantum computing and communication systems. By combining the strengths of nonlinear optics and nanophotonics, this work paves the way for advancing future quantum technologies.

## Supplementary information

Supplementary Information

## Data Availability

All data generated or analysed during this study are included in this published article [and its supplementary information files].
