# Supplementary material for: Nonlinear nanophotonics for high-dimensional quantum states
Source: Light Sci Appl. 2026 Jan 29;15:92. doi: 10.1038/s41377-025-02179-0 (PMC12852679; doi:10.1038/s41377-025-02179-0)
Supplement: Supplementary file 1 — Supplementary Information [file 41377_2025_2179_MOESM1_ESM.pdf]

Supplementary Information for Nonlinear Nanophotonics for High-Dimensional Quantum States

Section A: coupling a single photon to a nanophotonic platform:

The quantum state of angular momentum of light in general consists of polarization (spin), and orbital angular momentum (OAM). In our study, we couple photons using circular coupler with $l_m = 0\hbar$ and coupling out through circular coupler. Considering the nanophotonic quantum state in this scenario, we obtain the following:

When photons are confined to a metal-dielectric surface, they induce excitations in both in-plane field components. This results in an inseparable quantum state, intertwining the basis vectors of the spin and OAM degrees of freedom (DoFs). The deterministic relationships among the field components are governed by Maxwell's equations, ensuring that the Total Angular Momentum (TAM) remains identical across all components.

There are two conventional approaches for extending across the field components: one addresses its in-plane components X and Y, along with the out-of-plane component Z, and the other extends the vector fields through its in-plane left-handed (LH, σ_+) and right-handed (RH, σ_-) rotating components, in addition to its normal Z component.

Following the first approach, the basis in which the OAM remains orthogonal can be described using Hermite-Bessel (HB) modes - formed as a superposition of Bessel modes. In this representation, the OAM orthogonality is defined through the nth order Bessel modes.

The TAM of the confined photon is determined by summing all contributions from angular momentum, expressed as $J = L + S + l_m$, where l_m represents the helicity of the coupler. Without loss of generality, let us choose a coupler with $l_m = n \cdot \hbar$. The out-of-plane electric field component can be described as:

$$E_z(r, \theta, \phi) = E_0 J_n(k_{SPP} r) = \frac{E_0}{2\pi} \int_0^{2\pi} J_n e^{-i(k cos(\theta)x + k sin(\theta)y)} e^{in\theta} \tag{S1}$$

The in-plane components are described in both scenarios as:

$$\begin{pmatrix} E_{\sigma_+} \\ E_{\sigma_-} \end{pmatrix} = \begin{pmatrix} \frac{E_H + iE_V}{\sqrt{2}} \\ \frac{E_H - iE_V}{\sqrt{2}} \end{pmatrix} = \frac{E_0 \kappa}{k_{SPP}} \begin{pmatrix} -J_{n-1}(k_{SPP} r) \\ J_{n+1}(k_{SPP} r) \end{pmatrix}$$

$$= \frac{E_0 \kappa}{2\pi k_{SPP}} \begin{pmatrix} -\int_0^{2\pi} j_{n-1} e^{-i(k cos(\theta)x + k sin(\theta)y)} e^{i(n-1)\theta} \\ \int_0^{2\pi} j_{n+1} e^{-i(k cos(\theta)x + k sin(\theta)y)} e^{i(n+1)\theta} \end{pmatrix} \tag{S2}$$

$$\begin{pmatrix} E_H \\ E_V \end{pmatrix} = \frac{E_0\kappa}{2k_{SPP}} \begin{pmatrix} J_{n-1}(k_{SPP}r) - J_{n+1}(k_{SPP}r) \\ i\big(J_{n-1}(k_{SPP}r) + J_{n+1}(k_{SPP}r)\big) \end{pmatrix}$$

$$= \frac{E_0\kappa}{4\pi k_{SPP}} \begin{pmatrix} \int_0^{2\pi} j_{n-1} e^{-i(kcos(\theta)x+ksin(\theta)y)} e^{i(n-1)\theta} - \int_0^{2\pi} j_{n+1} e^{-i(kcos(\theta)x+ksin(\theta)y)} e^{i(n+1)\theta} \\ i\left(\int_0^{2\pi} j_{n-1} e^{-i(kcos(\theta)x+ksin(\theta)y)} e^{i(n-1)\theta} + \int_0^{2\pi} j_{n+1} e^{-i(kcos(\theta)x+ksin(\theta)y)} e^{i(n+1)\theta}\right) \end{pmatrix} \quad \text{(S3)}$$

in our study we coupled photons using Archimedean spiral coupler $l_m = 0\ \hbar$. Let us develop the specific nanophotonic quantum state associated with that case:

1. A left-handed polarized photon $|\sigma_-\rangle|j_0\rangle$ incident upon our nanophotonic system results in plasmon state which carry TAM of $-1\ \hbar$, $|J_{-1}\rangle$. Its mode components can be scattered through the coupler, generating a photon that displays entanglement between its SAM and OAM:

$$|J_{-1}\rangle = \frac{1}{\sqrt{2}}(|RH\rangle \otimes |j_0\rangle - |LH\rangle \otimes |j_{-2}\rangle) =$$

$$= \frac{1}{\sqrt{2}}\left(\left(\frac{|X\rangle - i|Y\rangle}{\sqrt{2}}\right) \otimes |j_0\rangle - \left(\frac{|X\rangle + i|Y\rangle}{\sqrt{2}}\right) \otimes |j_{-2}\rangle\right) =$$

$$= \frac{1}{\sqrt{2}}\left(|X\rangle \otimes \left(\frac{|j_0\rangle - |j_{-2}\rangle}{\sqrt{2}}\right) - i|Y\rangle \otimes \left(\frac{|j_0\rangle + |j_{-2}\rangle}{\sqrt{2}}\right)\right)$$

(S4)

2. A right-handed polarized photon $|\sigma_+\rangle|j_0\rangle$ incident upon our system results in plasmons with TAM state of $|J_1\rangle$ which carry $1\ \hbar$ TAM. Its mode components can be scattered through the coupler and be described as:

$$|J_1\rangle = \frac{1}{\sqrt{2}}(|RH\rangle \otimes |j_2\rangle - |LH\rangle \otimes |j_0\rangle) =$$

$$= \frac{1}{\sqrt{2}}\left(\left(\frac{|X\rangle - i|Y\rangle}{\sqrt{2}}\right) \otimes |j_2\rangle - \left(\frac{|X\rangle + i|Y\rangle}{\sqrt{2}}\right) \otimes |j_0\rangle\right) =$$

$$= \frac{1}{\sqrt{2}}\left(|X\rangle \otimes \left(\frac{|j_2\rangle - |j_0\rangle}{\sqrt{2}}\right) - i|Y\rangle \otimes \left(\frac{|j_2\rangle + |j_0\rangle}{\sqrt{2}}\right)\right)$$

(S5)

3. A horizontally polarized photon $|H\rangle|j_0\rangle$ incident upon our system results in plasmons with total angular momentum state of $\frac{|J_1\rangle+|J_{-1}\rangle}{\sqrt{2}}$ which carry superposition of $\pm 1\ \hbar$ TAM. Its mode components can be scattered through the coupler and be described as:

$$\frac{|J_1\rangle + |J_{-1}\rangle}{\sqrt{2}} = |RH\rangle \otimes \left(\frac{|j_2\rangle - |j_0\rangle}{\sqrt{2}}\right) + |LH\rangle \otimes \left(\frac{|j_{-2}\rangle - |j_0\rangle}{\sqrt{2}}\right) = \quad \text{(S6)}$$

$$= \frac{1}{\sqrt{2}}\left(\left(\frac{|X\rangle - i|Y\rangle}{\sqrt{2}}\right) \otimes \left(\frac{|j_2\rangle - |j_0\rangle}{\sqrt{2}}\right) + \left(\frac{|X\rangle + i|Y\rangle}{\sqrt{2}}\right) \otimes \left(\frac{|j_{-2}\rangle - |j_0\rangle}{\sqrt{2}}\right)\right) =$$

$$= \frac{1}{\sqrt{2}}\left(|X\rangle \otimes \left(\frac{|j_2\rangle + |j_{-2}\rangle}{\sqrt{2}}\right) - i|Y\rangle \otimes \left(\frac{|j_2\rangle - 2|j_0\rangle - |j_{-2}\rangle}{\sqrt{2}}\right)\right) =$$

$$= \frac{1}{\sqrt{2}}(|X\rangle \otimes |HB_{11}\rangle - i|Y\rangle \otimes |HB_{02}\rangle) =$$

$$= |RH\rangle \otimes \left(\frac{|HB_{11}\rangle + |HB_{02}\rangle}{\sqrt{2}}\right) + |LH\rangle \otimes \left(\frac{|HB_{11}\rangle - |HB_{02}\rangle}{\sqrt{2}}\right)$$

4. A vertically polarized photon $|V\rangle|j_0\rangle$ incident upon our system results in plasmons with total angular momentum state of $\frac{|J_1\rangle - |J_{-1}\rangle}{\sqrt{2}i}$ which carry superposition of $\pm 1\,\hbar$ TAM. Its mode components can be scattered through the coupler and describe as:

$$\frac{|J_1\rangle - |J_{-1}\rangle}{\sqrt{2}i} = |RH\rangle \otimes \left(\frac{|j_2\rangle - |j_0\rangle}{\sqrt{2}}\right) - |LH\rangle \otimes \left(\frac{|j_{-2}\rangle + |j_0\rangle}{\sqrt{2}}\right) =$$

$$= \frac{1}{\sqrt{2}}\left(\left(\frac{|X\rangle - i|Y\rangle}{\sqrt{2}}\right) \otimes \left(\frac{|j_2\rangle - |j_0\rangle}{\sqrt{2}}\right) - \left(\frac{|X\rangle + i|Y\rangle}{\sqrt{2}}\right) \otimes \left(\frac{|j_{-2}\rangle + |j_0\rangle}{\sqrt{2}}\right)\right) =$$

$$= \frac{1}{\sqrt{2}}\left(|X\rangle \otimes \left(\frac{-|j_2\rangle + 2|j_0\rangle + |j_{-2}\rangle}{\sqrt{2}}\right) - i|Y\rangle \otimes \left(\frac{|j_2\rangle + |j_{-2}\rangle}{\sqrt{2}}\right)\right) =$$

$$= \frac{1}{\sqrt{2}}(|X\rangle \otimes |HB_{20}\rangle - i|Y\rangle \otimes |HB_{11}\rangle) =$$

$$= |RH\rangle \otimes \left(\frac{|HB_{20}\rangle + |HB_{11}\rangle}{\sqrt{2}}\right) + |LH\rangle \otimes \left(\frac{|HB_{20}\rangle - |HB_{11}\rangle}{\sqrt{2}}\right)$$

(S7)

Section B: Nanophotonics-enhanced Quantum Key Distribution

In this section, we propose the use of the two qudits encoded in the far-field photons (depending on the pump polarization) for a quantum key distribution (QKD) protocol with the use of qudits instead of qubits. This allows the sender to encode each digit of the key by choosing one of the bases. A photon is then prepared in a specific polarization state corresponding to the digit value (see eq. 9 and 12 in the main text), with the selected basis defining the encoding scheme. Building on the well-known BB84 protocol [49,50], our scheme extends its framework to a higher-dimensional version by employing qudits instead of qubits. The BB84 protocol is a foundational QKD scheme designed to establish a secure communication channel between two parties, Alice and Bob, by exploiting the principles of quantum mechanics. By encoding information in qudits across two distinct bases, the proposed scheme not only generalizes the BB84 protocol but also offers the potential for enhanced key rate, as qudits carry more

information per photon compared to qubits [51]. This high-dimensional approach improves the efficiency of the protocol, demonstrating the utility of the generated qudits for advanced quantum communication applications.

Our approach utilizes the nanophotonic platform presented in Fig. 1 in the main text to generate a shared key based on the SAM and the OAM DoFs of the far-field photons released from the nanophotonic platform. As we show below, incorporating multiple DoFs allows to encode quantum information on multilevel bases, paving the way to enhanced coding density compared to standard protocols relying only on the binary polarization DoF (as in BB84).

In our protocol, Alice and Bob utilize a classical channel for public communications, through which they announce their respective choices of measurement bases for their photons. Due to the conservation of TAM and the predefined quantum states generated in the near field, Alice can predict the outcome of Bob's measurement once she measures her photon — unless an eavesdropper has interfered.

We now outline the QKD protocol step by step:

a. *Randomization*

In order to distill a secret key, we need to generate two random processes, comprising the sequence values and transmission bases.

In our realization, the basis selection simply switches between a circular and linear polarization of the pump field. The data sequence is obtained from a sequence of four-dimensional qudits, which are generated by two sequential binary random selections: one is the specific polarization handedness (direction) of the pump field, as explained above, and the second is the random selection of the photon TAM value which is performed by a beam splitter (see Fig. S1). The nanophotonic system stores two SPPs with different TAM as described in eq. 7. When extracted by the pump field they pass through a 50:50 beam splitter. At the output, one of the photons is directed to the communications channel (i.e., to Bob). and the other goes to Alice's measurement module. All other output combinations of the beam splitter are not viable for the key generation. Alice measures her photon and based on the measurement outcome, she deterministically knows which photon was sent to Bob. This measurement also allows Alice to herald and synchronize Bob's measurements.

The logical ququart states, $|0\rangle_L$, $|1\rangle_L$, and $|2\rangle_L$ and $|3\rangle_L$, which are associated with the digits of our secret key, are then encoded, as presented in the next section.

b. Encoding

As a result of the process – the photons sent to Bob are four valued qudits, which carry their information in their SAM and OAM DoFs. Depending on the pump basis (circularly / linearly polarized), and the basis of the biphoton state that is coupled to the nanophotonic sample, the output photon can be encoded in two different ways, as explained in the main text and presented in equations (9) and (12).

Overall, this gives us eight potential measurement outcomes for both Alice and Bob, and each of these outcomes represents one of the four possible digits in the secure key according to the encoding described above.

c. *Transmission*

Bob receives the transmitted photon and randomly selects one of the two measurement bases (circular or linear polarization). If the circular polarization is selected, he transfers the photon through a quarter waveplate followed by a polarization beam-splitter and, using a proper phase mask, the orbital angular momenta of the photons are detected: $\{|j_0\rangle, |j_2\rangle\}$ in the σ_- arm and $\{|j_{-2}\rangle, |j_0\rangle\}$ in the σ_+ arm. On the other hand, if the linear polarization basis is selected, Bob transfers the photon through a polarization beam splitter, and then, by using a matched amplitude filter, the photon with the proper Hermit Bessel order is detected: $\{|HB_{11}\rangle, |HB_{20}\rangle\}$ in the H arm and $\{|HB_{02}\rangle, |HB_{11}\rangle\rangle\}$ in the V arm (figure 3). The derivation of the possible modes achieved by the chosen mask is elaborated in the supplementary information. The successful transmission relies on the condition that **Alice and Bob must select identical bases for both the pump and the OAM mask used by Bob**.

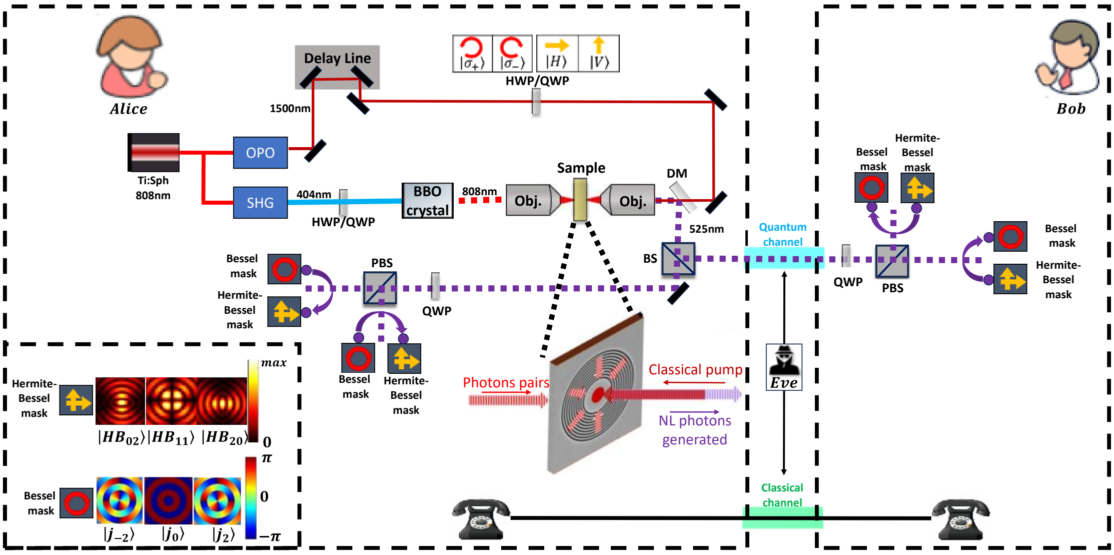

Fig. S1. Proposed Experimental Configuration for the Nanophotonic QKD Protocol: Alice utilizes a short pulse laser at 404 nm photons followed by an SPDC process in a BBO crystal, and an OPO to generate a 1500 nm pump. She launches the entangled biphoton state to the nanophotonic sample, giving rise to the quantum nanophotonic modes. Illumination of the sample from the opposite side with a classical pump, which is generated by OPO using the same pulsed laser source, initiates non-linear interactions within the nanophotonic field. This interaction leads to the emission of new photons, which one of them is measured by Alice and the other one is sent to Bob through a quantum channel. Bob selects a mask, which is matched either to Bessel or HB mode pattern, and detects the OAM of the photon. In addition, Bob and Alice share a classical channel for public discussion. The setup includes half-waveplate (HWP) and quarter-waveplate (QWP), beam splitter (BS) and dichroic mirror (DM) incorporated into the system.

d. *Public Discussion and Information Reconciliation*

Next, Alice and Bob publicly announce their selections: Alice reveals the pump polarization type (circular or linear), and Bob reveals the basis of his measurements (either circular or linear). They discard any instances where the basis does not match. From the remaining sub-sequence, they distill the secret key. Figure S2 depicts a truth table encompassing all possible measurements, assuming that the participants have selected identical bases.

Similar to the BB84 standard protocol, Alice refrains from publicly disclosing the entire state of the transmitted photon. She only declares the pump polarization and her initial biphoton state, without revealing the OAM she measured. Bob, from his side, shares the basis of the mask he employed to measure the OAM, but does not disclose the specific OAM he measured. This approach ensures that a

potential eavesdropper monitoring the public channel during the information reconciliation stage obtains only partial information.

e. *Error Check*

In the previous step, Alice and Bob publicly reveal and compare the choices Alice made for her pump and Bobs' basis mask. Bob can choose either one of two possible masks to measure the OAM: if he chooses a circular mask then the possible OAM states can be $\{|j_0\rangle, |j_2\rangle, |j_{-2}\rangle\}$ (see fig. 2(c-d) in the main text). If he chooses the linear basis, he can measure an OAM state from $\{|HB_{02}\rangle, |HB_{20}\rangle, |HB_{11}\rangle\}$ (see fig. 2(a-b). A discrepancy in the spatial distribution he observes, whether it is a measurement outcome that differs from the 4 possible outcomes, or a different basis compared to the basis Alice chose for her pump at the level of the public discussion, would indicate potential interference from an eavesdropper. In addition, since the two photons emitted from the nanophotonic sample are correlated (in the circular basis if one has a TAM J_1 then the other photon must have TAM J_{-1}, and in the linear basis the two photons must have the same TAM). In any case where Bob measured TAM different than these two conditions, he knows that an error has occurred. Additionally, by measuring his own photon, Bob can deduce what OAM state Alice has measured and vice versa.

f. *Secure Key Generation and Privacy Amplification*

At the end of the public discussion, Alice and Bob use the remaining string to distill a shared key. Two modes are formed in the nanophotonic platform. Alice measures one mode, and the other is transmitted to Bob. Leveraging the correlation between the two modes, Alice can infer the TAM J of the state of the photon transmitted to Bob, enabling the extraction of encoding and the generation of a secure key. A truth table illustrating the various potential states in which Alice and Bob can engage is presented in Figure S2.

g. *Quantum Bit Error Rate (QBER)*

Consider the raw key rate (in the absence of errors) $R_{raw} = f_r\eta(1 - e^{-\mu})$ where f_r is the pulse repetition frequency, μ is the average number of photons arriving, and η is the photodetector efficiency [54]. For a low number of photons, the factor $(1 - e^{-\mu})$ can be approximated by the fiber loss. Typical values are $f_r = 2.5$ GHz, $\eta = 0.9$, and 0.2 dB/km loss [55,56]. Having the raw key rate, one may estimate the QBER as QBER$=\frac{P_e}{R_{raw}+P_e}$, based on the error rate P_e, $P_e = P_{dark} + P_a$, where P_{dark} and P_a are the probabilities of a dark count event and a false after-pulse count, respectively. Typical values are 1000 per second at a cryogenic temperature, and $P_a = 0.02$ [55]. By using Law Density Parity Check (LDPC) error correction codes at rate $R_{coding} = 0.6$, one may achieve QBER of $\leq 6\%$ [54]. This guarantees that there is no leakage to Eve.

h. *Enhanced Key Rate in Noisy Channels*

QKD relies on quantum properties to detect eavesdropping, with the quantum key rate quantifying the rate of secure bit generation. A positive key rate ensures secret key distribution despite noise and attacks. The QBER is the ratio of the error to the key rate, and can indicate potential information leakage. High QBER suggests strong noise, possibly revealing Eve's presence.

As we show next, our approach of utilizing qudits enhances the key rate. To show that, we analyze two noisy channels. The first is a quantum erasure channel, where each transmitted qudit is either received intact (probability $1 - \mathcal{E}$) or lost ($\mathcal{E}$). For an n-level system, the key rate follows $R = (1 - 2\mathcal{E}) \log_2 n$. With 4-level qudits, this results in $R = 2(1 - 2\mathcal{E})$ for $0 \leq \mathcal{E} \leq \frac{1}{2}$. If more than half the photons are lost ($\mathcal{E} \geq \frac{1}{2}$), the key rate drops to zero. Using qudits instead of qubits doubles the key rate compared to standard QKD [54,57,58], as we elaborate in the supplementary material.

The second channel we developed and analyzed, models OAM state transitions as a generalized amplitude damping channel. Here, received the quantum states may undergo transitions between OAM states, governed by three distinct probabilities: p_1 (transition from j_0 to j_2 or vice versa), p_2 (from j_{-2} to j_0 or vice versa), and p_3 (from j_{-2} to j_2 or vice versa). These probabilities are complementary, assuming that the fiber describing the quantum channel supports only these three OAM modes. These transitions introduce errors that affect the security and efficiency of the key distribution. We provide a detailed characterization of this channel and its impact on the key rate in the supplementary material. The resulting key rate expression for this noisy channel is more complex, as it depends on the entropy terms and the probabilities p_1, p_2, p_3, reflecting the effect of amplitude damping and state mixing. The QBER in this case is primarily determined by the probability of unwanted state transitions in the damping channel and any residual errors in the measurement process. It will depend on how frequently these transitions occur. If the probabilities $\{p_1, p_2, p_3\}$ are small, errors remain limited, and the key rate advantage from qudits persists.

Unlike the erasure channel, where the key rate scales linearly with the erasure probability $\mathcal{E}$, the key rate in our noisy channel depends on the probability distributions of state transitions, introducing a more intricate dependence on the noise characteristics (see derivation in supplementary material). However, for low-noise scenarios (small p_1, p_2, p_3), our method retains a significant key rate advantage due to the qudit encoding- akin to the erasure channel, where increasing the digit count logarithmically boosts the key rate compared to a standard QKD protocol using qubits (two-level systems).

To evaluate the total key generation rate, we also account for the photon pair generation rate. The photon generation efficiency in our nanophotonic chip, based on four-wave mixing in a gold-dielectric structure, is influenced by absorption and transmission properties. Based on a previous work with those nanophotonic chips [24], specifically in this case, a significant portion of the pump power is retained, supporting efficient photon pair generation while balancing loss. Additionally, achieving the desired plasmonic mode for the qudit generation requires ensuring that the nonlinear interaction consistently leads to the emission of exactly two single photons into the far field, originating from the nanophotonic modes discussed in Section 4. This process is governed by the pump intensity. The nonlinear interaction involves two pulses, and by carefully synchronizing their intensities, each pulse pair can be tuned to generate precisely two photons. Consequently, the qudit generation rate is determined by the pump intensity, which, in turn, dictates the formation of plasmonic modes that encode the qudits in the two emitted photons from the nanophotonic platform.

Thus, the overall secure bit rate, measured in bits per second, is given by: $R_{total} = R \times G$, where R is the key rate (bits per photon) and G is the photon generation rate (photons per second). By increasing

both the qudit dimensionality and optimizing the photon generation with the intensity of the pump, the key rate in our method increases.

Section C: Key rate analysis

The basic idea of QKD is to use the properties of quantum states to detect any eavesdropping attempts. **The quantum key rate is a measure of the rate at which qubits can be reliably exchanged between the sender (Alice) and the receiver (Bob) in a QKD protocol.** The quantum key rate essentially represents the secure key bits generated per channel use, considering the efficiency of various processes involved in QKD. Achieving a positive quantum key rate indicates that Alice and Bob can distill a secret key despite the presence of noise and potential eavesdropping.

The encryption key rate is defined as $R = \lim_{N\to\infty} \frac{\ell(N)}{N}$, where N is the number of iterations, and $\ell(N)$ stands for the length of the resulting key sequence. Based on information-theoretic consideration, QKD attains the key rate [1]

$$R = I(X;Y) - I(X;E)_\rho \qquad \text{(S8)}$$

$I(A;B)_\rho$ denotes the quantum mutual information between A and B, with respect to a joint state ρ_{AB}. Specifically, the quantum mutual information is defined as $I(A;B)_\rho = H(\rho_A) + H(\rho_B) - H(\rho_{AB})$ where $H(\rho) = -Tr[\rho \log \rho]$ is the Von Neuman entropy.

Intuitively, the mutual information measures the correlation between A and B. The symbols X and Y represent the classical encodings employed by Alice and Bob in their communication, respectively. Moreover, X represents the symbol that Alice intends to transmit to Bob, i.e., the digit that would have

**Alice random classical pump**	$\|\sigma_-\rangle$	$\|\sigma_-\rangle$	$\|\sigma_+\rangle$	$\|\sigma_+\rangle$	$\|H\rangle$	$\|V\rangle$	$\|H\rangle$	$\|V\rangle$
**Alice's OAM measurement**	j_0	j_2	j_0	j_{-2}	HB_{20}	HB_{11}	HB_{11}	HB_{02}
**TAM state of Alice**	J_{-1}	J_1	J_1	J_{-1}	J_+	J_+	J_-	J_-
**Alice's secret key**	**3**	**2**	**0**	**1**	**1**	**2**	**3**	**0**
**Bob's random mask**								
**Bob's OAM measurement**	j_2	j_0	j_{-2}	j_0	HB_{20}	HB_{11}	HB_{11}	HB_{02}
**TAM state of Bob**	J_1	J_{-1}	J_{-1}	J_1	J_+	J_+	J_-	J_-
**Public discussion**								
**Shared secret key**	**3**	**2**	**0**	**1**	**1**	**2**	**3**	**0**

Fig. S2. Truth table of all possible digits in the cryptographic key using the nanophotonic QKD protocol. Each participant, including Alice and Bob, may have multiple potential digits in their respective keys. The secret key generation entails publicly declaring their measurements. The brown frame represents the public discussion (via a classical channel) from Alice and Bob's side – Alice declares her pump polarization, and Bob declares his random measurement mask. If they measure in different bases, akin to the BB84 protocol, the procedure is halted. Conversely, if their measurements align in the same basis, both Alice and Bob can deduce the exclusive secret digit they share. The green frame represents the quantum channel, where the photon emitted from the nanophotonic sample is transmitted to Bob.

been shared between Alice and Bob. Hence, X represents both the single photon and the classical pump sent on the nanophotonic chip. Y represents the digit that Bob measures from the noisy channel output. $\ell(N)$ is the number of digits in the generated key. The Holvo information, terms $I(X;Y)$ and $I(X;E)_\rho$ can be interpreted as the rate of information that reaches Bob and Eve, respectively, and the substruction of $I(X;E)_\rho$ can be viewed as the rate sacrifice that is needed for security.

In our protocol, we analyze two noisy channels: first, the quantum erasure channel – where a transmitter sends a qudit, and the receiver either receives the original qudit state (i.e., Bob receives the photon passed through the quantum channel), with probability $\mathbf{1-\varepsilon}$, or an erasure (i.e., the photon is lost) the state with some probability. The second channel model we analyze can be viewed as a generalized amplitude damping channel [2]. In certain instances, the received quantum states may be in superpositions. The decay is captured by the probability amplitudes of specific states in the amplitude damping channel model, resulting in information loss upon measurement. This phenomenon resembles classical communication channels where interference can attenuate signal power.

In the formulated noisy channel, three distinct probabilities govern the conversion of OAM states. Without loss of generality, we describe the physical channel using the circular basis, although the same arguments apply to the linear basis. Within the circular basis, one of three possible OAM states $\{|j_0\rangle, |j_2\rangle, |j_{-2}\rangle\}$ can be measured. P_1 represents the probability that an OAM state $|j_0\rangle$ at the input will transform into OAM state $|j_2\rangle$ at the output, and vice versa. Similarly, P_2 signifies the probability of the transformation between $|j_0\rangle$ and $|j_{-2}\rangle$, and vice versa, while P_3 denotes the probability of transformation between $|j_2\rangle$ and $|j_{-2}\rangle$, and vice versa. These probabilities are complementary, assuming that the fiber describing the quantum channel supports only these three OAM modes.

The key rate for this quantum channel is determined utilizing tools of quantum information theory such as Kraus operators.

<u>Key Rate Analysis in the Presence of an Erasure Channel</u>

We first examine the key rate in the standard BB84 protocol, where the secured key has 2 digits, for the erasure channel model. Next, we examine the key rate in a case where there are 4 possible digits. We show that adding more digits in the key enhances the key rate logarithmically: for n digits, the key rate becomes $R = (1-2\varepsilon)\log_2 \text{n}$. Following this, we proceed to showcase specifically that the key rate for our nanophotonic QKD protocol can be determined similarly. We assume an erasure channel on the emitted photon that Alice sent to Bob. The key rate in our case has the value of a quaternary QKD protocol with 4 digits:

$$R = (1-2\varepsilon)\log_2 4 = 2 - 4\varepsilon \qquad \text{(S9)}$$

for $0 \le \varepsilon \le \frac{1}{2}$, where ε is the erasure probability. Otherwise, if $\frac{1}{2} \le \varepsilon \le 1$, more than half the photons are lost to the environment, hence the key rate is $R = 0$. Namely, we doubled the key rate compared to the standard binary protocol.

We analyze the key rate of the BB84 protocol under an erasure channel, showing that increasing from 2 to 4 digits improves the key rate. We then demonstrate that a similar approach applies to our Spin-Orbit QKD protocol.

In general, a key rate of length $\ell(N)$ defined as:

$$R = \lim_{N\to\infty} \frac{\ell(N)}{N} = I(X;Y)_\rho - I(X;E)_\rho \quad \text{(S10)}$$

Where $I(X;Y)$ is the classical mutual information, which is interpreted as the amount of information, which is interpreted as the amount of information that the random variable X and Y share. The Holevo information term, $I(X,E)_\rho$, can be thought of as the rate of information leakage to the environment. Here, Y stands for Bob's measurement outcome, and E represents Eve's system (the environment).

The erasure channel is defined as follows:

$$\mathcal{N}_{A\to B}(\rho) = (1-\varepsilon)\rho + \varepsilon|e\rangle\langle e| \quad \text{(S11)}$$

Every quantum channel $\mathcal{N}_{A\to B}$ has a Stinespring representation: $\mathcal{N}_{A\to B}(\rho) = \mathrm{Tr}_E(V\rho V^\dagger)$, where V is an isometry, i.e. $V^\dagger V = \mathbb{I}_A$. For the erasure channel, $V = \sqrt{1-\varepsilon}\mathbb{I}_{A\to B} \otimes |e\rangle_E + \sqrt{\varepsilon}|e\rangle_B \otimes \mathbb{I}_{A\to E}$. Bob's output can be extended as follows:

$$\sigma_{ZB} = (1-\varepsilon)|0\rangle\langle 0| \otimes \rho + \varepsilon|1\rangle\langle 1| \otimes |e\rangle\langle e| \quad \text{(S12)}$$

Where Z is a classical flag that indicates whether an erasure (loss) has occurred, for every input density operator ρ on the Hilbert space $\mathcal{H}_A$, where the operator $V: \mathcal{H}_A \to \mathcal{H}_B \otimes \mathcal{H}_E$ satisfies $V^\dagger V = \mathbb{I}$. We refer to the systems A, B, and E as belonging to Alice, Bob, and Eve (i.e., the eavesdropper), respectively. Z is an indicator which gives 0 if there wasn't erasing, and 1 if an erasure has occurred. The erasure state $|e\rangle$ is orthogonal to the 2 transmitted qudits, hence $\mathcal{H}_B = \mathcal{H}_E = \mathbb{C}^{\{0,1,2,3,e\}}$. That is, Bob and Eve's Hilbert spaces are of dimension 5.

Now, as each of the four potential digits possesses an equal distribution: $P_x = \left(\frac{1}{4}, \frac{1}{4}, \frac{1}{4}, \frac{1}{4}\right)$, given $x = X$ for $x \in \{0,1,2\}$, the density matrix is:

$$\rho_{ZB}^x = (1-\varepsilon)|0\rangle\langle 0| \otimes |x\rangle\langle x| + \varepsilon|1\rangle\langle 1| \otimes |e\rangle\langle e| \quad \text{(S13)}$$

and the conditional entropy of Bob's measurement outcome is: $H(Y|X)_\rho = H(\varepsilon, 1-\varepsilon)$

However, without conditioning on x, the density operator is:

$$\rho_{ZB} = (1-\varepsilon)|0\rangle\langle 0| \otimes \left[\frac{1}{4}|0\rangle\langle 0| + \frac{1}{4}|1\rangle\langle 1| + \frac{1}{4}|2\rangle\langle 2| + \frac{1}{4}|3\rangle\langle 3|\right] + \varepsilon|1\rangle\langle 1| \otimes |e\rangle\langle e| \quad \text{(S14)}$$

Thus, the entropy:

$$H(B)_\rho = H(ZB)_\rho \equiv H(Z) + H(B|Z)_\rho = H(Z) + \sum_{z\in\{0,1\}} \Pr(Z=z)\cdot H(B|Z=z)$$

$$= H(1-\varepsilon,\varepsilon) + (1-\varepsilon)$$

$$\cdot H\left(\frac{1}{4}\Big(\underbrace{|0\rangle\langle 0| + |1\rangle\langle 1| + |2\rangle\langle 2| + |3\rangle\langle 3|}_{orthogonal\ states-spectral\ decomposition}\Big)\right)$$

$$+\ \varepsilon \quad \underbrace{H(|e\rangle\langle e|)}_{entropy\ of\ a\ pure\ state=0}$$

$$= H(1-\varepsilon,\varepsilon) + (1-\varepsilon)H\left(\frac{1}{4},\frac{1}{4},\frac{1}{4},\frac{1}{4}\right) + \varepsilon\cdot 0 \tag{S15}$$

Given that the von Neumann entropy with respect to the density operator ρ is expressed as $H(\rho) = -\mathrm{Tr}(\rho\log(\rho))$ we obtain: $H(B)_\rho = H(1-\varepsilon,\varepsilon) + (1-\varepsilon)\log_2 4$. Thus,

$$I(X;Y)_\rho = I(X;B)_\rho = H(B)_\rho - H(B|X)_\rho$$
$$= H(1-\varepsilon,\varepsilon) + (1-\varepsilon)\log_2 4 - H(1-\varepsilon,\varepsilon) = (1-\varepsilon)\log_2 4$$
$$= 2(1-\varepsilon). \tag{S16}$$

Our current focus is on computing the Holevo information term, $I(X;E)_\rho$, where this classical-quantum mutual information captures the information that Eve gains on Alice's raw key. The Holevo information is defined in terms of entropies (denoted as H): $I(X;E)_\rho \equiv H(E)_\rho - H(E|X)_\rho$. Since the complementary channel, from Alice (A) to Eve (E), is an erasure channel with an erasure probability $\bar{\varepsilon} = 1-\varepsilon$, we have

$$I(X;E)_\rho = \big(1-(1-\varepsilon)\big)\log_2 4 = 2\varepsilon \tag{S17}$$

Therefore, we can calculate the entire key rate. Using equation (S8), the key rate R:

$$R = I(X;Y) - I(X;E)_\rho = \underbrace{2(1-\varepsilon)}_{I(X;B)_\rho} - \underbrace{2\varepsilon}_{I(X;E)_\rho} = 2(1-2\varepsilon) \tag{S18}$$

<u>Nanophotonics QKD - parameters and definitions:</u>

We define the density operator according to the uniform distribution of all the digits in the key. Notations: Alice has bipartite states $|\psi\rangle$, written in two possible bases (linear and circular bases):

$$|\psi\rangle = \frac{1}{\sqrt{2}}(|\sigma_+\rangle|\sigma_-\rangle + |\sigma_-\rangle|\sigma_+\rangle) = \frac{1}{\sqrt{2}}(|H\rangle|H\rangle - |V\rangle|V\rangle)$$

In addition, she has a classical pump either in a linear basis or in a circular basis $|\sigma_+\rangle_{A_1}/|\sigma_-\rangle_{A_1}/|H\rangle_{A_1}/|V\rangle_{A_1}$.

After sending her state to the nanophotonic platform, a sum frequency generation is occurring, and two photons are emitted. Their spin is determined by the classical pump of Alice, and its OAM is determined according to the spin-orbit coupling occurred in the nanophotonic platform. Alice measured one photon and the second one sent to Bob.

The joint density matrix that describes the joint state of Alice and Bob is given by

$$\rho_{A_1,A_2,B_1,B_2} = \frac{1}{8}|\sigma_+\rangle_{A_1}\langle\sigma_+|_{A_1}\otimes|j_0\rangle_{A_2}\langle j_0|_{A_2}\otimes|\sigma_+\rangle_{B_1}\langle\sigma_+|_{B_1}\otimes|j_{-2}\rangle_{B_2}\langle j_{-2}|_{B_2} \quad \text{"0"}$$

$$+\frac{1}{8}|\sigma_+\rangle_{A_1}\langle\sigma_+|_{A_1}\otimes|j_{-2}\rangle_{A_2}\langle j_{-2}|_{A_2}\otimes|\sigma_+\rangle_{B_1}\langle\sigma_+|_{B_1}\otimes|j_0\rangle_{B_2}\langle j_0|_{B_2} + \quad \text{"1"}$$

$$+\frac{1}{8}|\sigma_-\rangle_{A_1}\langle\sigma_-|_{A_1}\otimes|j_2\rangle_{A_2}\langle j_2|_{A_2}\otimes|\sigma_-\rangle_{B_1}\langle\sigma_-|_{B_1}\otimes|j_0\rangle_{B_2}\langle j_0|_{B_2} + \quad \text{"2"}$$

$$+\frac{1}{8}|\sigma_-\rangle_{A_1}\langle\sigma_-|_{A_1}\otimes|j_0\rangle_{A_2}\langle j_0|_{A_2}\otimes|\sigma_-\rangle_{B_1}\langle\sigma_-|_{B_1}\otimes|j_2\rangle_{B_2}\langle j_2|_{B_2} + \quad \text{"3"}$$

$$+\frac{1}{8}|V\rangle_{A_1}\langle V|_{A_1}\otimes|HB_{02}\rangle_{A_2}\langle HB_{02}|_{A_2}\otimes|V\rangle_{B_1}\langle V|_{B_1}\otimes|HB_{02}\rangle_{B_2}\langle HB_{02}|_{B_2} + \quad \text{"0"} \qquad \text{(S19)}$$

$$+\frac{1}{8}|H\rangle_{A_1}\langle H|_{A_1}\otimes|HB_{20}\rangle_{A_2}\langle HB_{20}|_{A_2}\otimes|H\rangle_{B_1}\langle H|_{B_1}\otimes|HB_{20}\rangle_{B_2}\langle HB_{20}|_{B_2} + \quad \text{"1"}$$

$$+\frac{1}{8}|V\rangle_{A_1}\langle V|_{A_1}\otimes|HB_{11}\rangle_{A_2}\langle HB_{11}|_{A_2}\otimes|V\rangle_{B_1}\langle V|_{B_1}\otimes|HB_{11}\rangle_{B_2}\langle HB_{11}|_{B_2} + \quad \text{"2"}$$

$$+\frac{1}{8}|H\rangle_{A_1}\langle H|_{A_1}\otimes|HB_{11}\rangle_{A_2}\langle HB_{11}|_{A_2}\otimes|H\rangle_{B_1}\langle H|_{B_1}\otimes|HB_{11}\rangle_{B_2}\langle HB_{11}|_{B_2} \quad \text{"3"}$$

Where A_1 and A_2 correspond to Alice's initial states – the classical pump polarization and the OAM of the emitted photon, respectively; B_1, B_2 corresponds to Bob's photon polarization and the measurement of the OAM, respectively. Each term in the expression can yield a different digit value for the key sequence. In other words, there are 8 potential measurement outcomes for both Alice and Bob, and each of these 8 outcomes represents one of the 4 possible digits in the secure key according to the encoding described above. A truth table describing the density matrix of all the possible states is given in Fig. S2. The classification for digits is determined according to the joint state of Alice and Bob since only after Alice and Bob communicate classically and publicly, they are able to generate the secured key.

Notice that there is equal change to get each of the digits in the key. As defined previously, a key rate of length $\ell(N)$ defined as:

$$R = \lim_{N\to\infty}\frac{\ell(N)}{N} = I(X;Y)_\rho - I(X;E)_\rho = H(X|E)_\rho - H(X|Y)_\rho \qquad \text{(S20)}$$

Where $H(X|E)_\rho$ and $H(X|Y)_\rho$ are the conditional von-Neumann entropies of Alice's key, considering Eve's knowledge and Bob's measurement outcomes, respectively. the entropies can be expressed also as: $H(X|Y)_\rho = H(XY)_\rho - H(Y)_\rho$. N is the number channel uses.

Assuming an indicator Z (where it equals to 0 if there was no erasure, and 1 if an erasure has occurred), we can write the entire density operator of Alice and Bob as:

$$\omega^{z=0}_{X,Y,A_1,A_2,B} = \frac{1}{4}\big(|00\rangle_{XY}\langle 00|_{XY}\otimes\rho^{X=Y=0}_{A_1,A_2,B_1,B_2} + |11\rangle_{XY}\langle 11|_{XY}\otimes\rho^{X=Y=1}_{A_1,A_2,B_1,B_2} + |22\rangle_{XY}\langle 22|_{XY}\otimes\rho^{X=Y=2}_{A_1,A_2,B_1,B_2} + |33\rangle_{XY}\langle 33|_{XY}\otimes\rho^{X=Y=3}_{A_1,A_2,B_1,B_2}\big)$$

$$\omega^{z=1}_{X,Y,A_1,A_2,B} = |ee\rangle_{XY}\langle ee|_{XY}\otimes\rho^{X=Y=e}_{A_1,A_2,B_1,B_2} = |ee\rangle_{XY}\langle ee|_{XY}\otimes\left(\frac{1}{8}|p\rangle_{A_1}\langle p|_{A_1} + |L\rangle_{A_2}\langle L|_{A_2}\right)\otimes|e\rangle_{B_1}\langle e|_{B_1}\otimes|e\rangle_{B_2}\langle e|_{B_2} \qquad \text{(S21)}$$

Where $\rho^{X=Y=0}_{A_1,A_2,B_1,B_2}$,$\rho^{X=Y=1}_{A_1,A_2,B_1,B_2}$,$\rho^{X=Y=2}_{A_1,A_2,B_1,B_2}$,$\rho^{X=Y=e}_{A_1,A_2,B_1,B_2}$, $\rho^{X=Y=3}_{A_1,A_2,B_1,B_2}$ are density operators derived from partial trace on ρ_{A_1,A_2,B_1,B_2}:

$$\begin{aligned}
\rho^{X=Y=0}_{A_1,A_2,B_1,B_2} = & \frac{1}{8}|\sigma_+\rangle_{A_1}\langle\sigma_+|_{A_1}\otimes|j_0\rangle_{A_2}\langle j_0|_{A_2}\otimes|\sigma_+\rangle_{B_1}\langle\sigma_+|_{B_1}\otimes|j_{-2}\rangle_{B_2}\langle j_{-2}|_{B_2} \\
& +\frac{1}{8}|V\rangle_{A_1}\langle V|_{A_1}\otimes|HB_{02}\rangle_{A_2}\langle HB_{02}|_{A_2} \\
& \otimes|V\rangle_{B_1}\langle V|_{B_1}\otimes|HB_{02}\rangle_{B_2}\langle HB_{02}|_{B_2}
\end{aligned}$$

$$\begin{aligned}
\rho^{X=Y=1}_{A_1,A_2,B_1,B_2} = & \frac{1}{8}|\sigma_+\rangle_{A_1}\langle\sigma_+|_{A_1}\otimes|j_{-2}\rangle_{A_2}\langle j_{-2}|_{A_2}\otimes|\sigma_+\rangle_{B_1}\langle\sigma_+|_{B_1}\otimes|j_0\rangle_{B_2}\langle j_0|_{B_2} \\
& +\frac{1}{8}|H\rangle_{A_1}\langle H|_{A_1}\otimes|HB_{20}\rangle_{A_2}\langle HB_{20}|_{A_2} \\
& \otimes|H\rangle_{B_1}\langle H|_{B_1}\otimes|HB_{20}\rangle_{B_2}\langle HB_{20}|_{B_2}
\end{aligned}$$

$$\begin{aligned}
\rho^{X=Y=2}_{A_1,A_2,B_1,B_2} = & \frac{1}{8}|\sigma_-\rangle_{A_1}\langle\sigma_-|_{A_1}\otimes|j_2\rangle_{A_2}\langle j_2|_{A_2}\otimes|\sigma_-\rangle_{B_1}\langle\sigma_-|_{B_1}\otimes|j_0\rangle_{B_2}\langle j_0|_{B_2} \\
& +\frac{1}{8}|V\rangle_{A_1}\langle V|_{A_1}\otimes|HB_{11}\rangle_{A_2}\langle HB_{11}|_{A_2} \\
& \otimes|V\rangle_{B_1}\langle V|_{B_1}\otimes|HB_{11}\rangle_{B_2}\langle HB_{11}|_{B_2}
\end{aligned}$$

$$\begin{aligned}
\rho^{X=Y=3}_{A_1,A_2,B_1,B_2} = & \frac{1}{8}|\sigma_-\rangle_{A_1}\langle\sigma_-|_{A_1}\otimes|j_0\rangle_{A_2}\langle j_0|_{A_2}\otimes|\sigma_-\rangle_{B_1}\langle\sigma_-|_{B_1}\otimes|j_2\rangle_{B_2}\langle j_2|_{B_2} \\
& +\frac{1}{8}|H\rangle_{A_1}\langle H|_{A_1}\otimes|HB_{11}\rangle_{A_2}\langle HB_{11}|_{A_2} \\
& \otimes|H\rangle_{B_1}\langle H|_{B_1}\otimes|HB_{11}\rangle_{B_2}\langle HB_{11}|_{B_2}
\end{aligned}$$

$$\begin{aligned}
\rho^{X=Y=e}_{A_1,A_2,B} = & \Big(\frac{1}{8}|\sigma_+\rangle_{A_1}\langle\sigma_+|_{A_1}\otimes|j_0\rangle_{A_2}\langle j_0|_{A_2}+\frac{1}{8}|V\rangle_{A_1}\langle V|_{A_1}\otimes|HB_{02}\rangle_{A_2}\langle HB_{02}|_{A_2} \\
& +\frac{1}{8}|\sigma_+\rangle_{A_1}\langle\sigma_+|_{A_1}\otimes|j_{-2}\rangle_{A_2}\langle j_{-2}|_{A_2}+\frac{1}{8}|H\rangle_{A_1}\langle H|_{A_1}\otimes|HB_{20}\rangle_{A_2}\langle HB_{20}|_{A_2} \\
& +\frac{1}{8}|\sigma_-\rangle_{A_1}\langle\sigma_-|_{A_1}\otimes|j_2\rangle_{A_2}\langle j_2|_{A_2}+\frac{1}{8}|V\rangle_{A_1}\langle V|_{A_1}\otimes|HB_{11}\rangle_{A_2}\langle HB_{11}|_{A_2} \\
& +\frac{1}{8}|\sigma_-\rangle_{A_1}\langle\sigma_-|_{A_1}\otimes|j_0\rangle_{A_2}\langle j_0|_{A_2}+\frac{1}{8}|H\rangle_{A_1}\langle H|_{A_1}\otimes|HB_{11}\rangle_{A_2}\langle HB_{11}|_{A_2}\Big) \\
& \otimes|e\rangle_{B_1}\langle e|_{B_1}\otimes|e\rangle_{B_2}\langle e|_{B_2}
\end{aligned}$$

(S22)

Thus, the mutual information:

$$I(X;Y)_\omega = H(Y) - H(Y|X) = H(ZY) - H(Y|X)$$

$$I(X;Y)_\omega = H(1-\varepsilon,\varepsilon) + (1-\varepsilon)\log_2 4 - H(1-\varepsilon,\varepsilon) = 2(1-\varepsilon)$$

$$I(X;E)_\omega = 1 - 2(1-\varepsilon) = 2\varepsilon$$

Thus, the key rate: $R = 2(1-\varepsilon) - 2\varepsilon = 2(1-2\varepsilon)$

Key rate analysis in the presence of a generalized amplitude damping channel

As defined the key rate of length $\ell(N)$ is defined as equation (S20).

In this section, we analyze the key rate for a new noisy channel. We analyze the channel for the Bessel case (the HB case is analogous to that analysis with different probabilities). The entire density matrix that describes the joint state that transfer through the channel between Alice and Bob without noise is:

$$\rho_{A_1,A_2,B_1,T} = \frac{1}{4}|\sigma_+\rangle_{A_1}\langle\sigma_+|_{A_1} \otimes |j_0\rangle_{A_2}\langle j_0|_{A_2} \otimes |\sigma_+\rangle_{B_1}\langle\sigma_+|_{B_1} \otimes |j_{-2}\rangle_T\langle j_{-2}|_T \quad \text{"0"}$$

$$+\frac{1}{4}|\sigma_+\rangle_{A_1}\langle\sigma_+|_{A_1} \otimes |j_{-2}\rangle_{A_2}\langle j_{-2}|_{A_2} \otimes |\sigma_+\rangle_{B_1}\langle\sigma_+|_{B_1} \otimes |j_0\rangle_T\langle j_0|_T + \quad \text{"1"}$$

$$+\frac{1}{4}|\sigma_-\rangle_{A_1}\langle\sigma_-|_{A_1} \otimes |j_2\rangle_{A_2}\langle j_2|_{A_2} \otimes |\sigma_-\rangle_{B_1}\langle\sigma_-|_{B_1} \otimes |j_0\rangle_T\langle j_0|_T + \quad \text{"2"} \qquad \text{(S23)}$$

$$+\frac{1}{4}|\sigma_-\rangle_{A_1}\langle\sigma_-|_{A_1} \otimes |j_0\rangle_{A_2}\langle j_0|_{A_2} \otimes |\sigma_-\rangle_{B_1}\langle\sigma_-|_{B_1} \otimes |j_2\rangle_T\langle j_2|_T \quad \text{"3"}$$

While A_1corresponds to the classical pump she selects and A_2 correspond to her OAM mesurement, B_1 correspond to the Bob photon polarization and T denotes the OAM of the photon emitted from the nonlinear process that is transmitted to Bob. For simplicity, we have designated the OAM states transmitted to Bob trough the noisy channel as follows: $|j_0\rangle_T = |0\rangle, |j_2\rangle_T = |1\rangle, |j_{-2}\rangle_T = |2\rangle$. The right column represents the digit the correspond the coding of the key, which we elaborate about in the main text.

Next, we define the Kraus operators in our channel:

$$K_1 = \sqrt{p_1}|1\rangle\langle 0|\,, K_2 = \sqrt{p_2}|2\rangle\langle 0|\,, K_3 = \sqrt{p_3}|2\rangle\langle 1|\,,$$

$$K_4 = \sqrt{p_1}|0\rangle\langle 1|\,, K_5 = \sqrt{p_2}|0\rangle\langle 2|\,, K_6 = \sqrt{p_3}|1\rangle\langle 2|\,,$$

$$K_7 = \sqrt{1-p_1-p_2}|0\rangle\langle 0| + \sqrt{1-p_1-p_3}|1\rangle\langle 1| + \sqrt{1-p_2-p_3}|2\rangle\langle 2|$$

The quantum channel is defined as follows:

$$\mathcal{N}_{T\to B_2}(\rho) = \sum_j K_j \rho K_j^\dagger \qquad \text{(S24)}$$

Note that thus operators which defined satisfy the necessary conditions for Kraus operators.

Every quantum channel $\mathcal{N}_{T\to E}$ has a Stinespring representation: $\mathcal{N}_{A\to T}(\rho) = \mathrm{Tr}_E(V\rho V^\dagger)$ where $V = \sum_j K_j \otimes |j\rangle_E$.

Thus:

$$\mathcal{N}_{T\to B_2}(\rho) = \mathrm{Tr}_E(V\rho V^\dagger) = \sum_{j=1}^{7} \mathrm{Tr}_E\Big(\big(K_j \otimes |j\rangle_E\big)\rho\big(K_j \otimes |j\rangle_E\big)^\dagger\Big)$$

$$= \sum_{j=1}^{7} \mathrm{Tr}_E\Big(\big(K_j\rho K_j^\dagger \otimes |j\rangle_E\langle j|_E\big)\Big)$$

(S25)

The density matrix:

$$
\begin{aligned}
\rho_{A_1,A_2,B_1,E} = \left(\mathbb{1}\otimes \mathcal{N}^{\mathrm{C}}_{T\to B_2}\right)\rho_{A_1,A_2,B_1,T} \\
&= \frac{1}{4}|\sigma_+\rangle_{A_1}\langle\sigma_+|_{A_1}\otimes|j_0\rangle_{A_2}\langle j_0|_{A_2}\otimes|\sigma_+\rangle_{B_1}\langle\sigma_+|_{B_1} \\
&\otimes\sum_{j=1}^{7}\left\|K_j|2\rangle\right\|^2|j\rangle_E\langle j|_E \\
&+\frac{1}{4}|\sigma_+\rangle_{A_1}\langle\sigma_+|_{A_1}\otimes|j_{-2}\rangle_{A_2}\langle j_{-2}|_{A_2}\otimes|\sigma_+\rangle_{B_1}\langle\sigma_+|_{B_1} \\
&\otimes\sum_{j=1}^{7}\left\|K_j|0\rangle\right\|^2|j\rangle_E\langle j|_E \\
&+\frac{1}{4}|\sigma_-\rangle_{A_1}\langle\sigma_-|_{A_1}\otimes|j_2\rangle_{A_2}\langle j_2|_{A_2}\otimes|\sigma_-\rangle_{B_1}\langle\sigma_-|_{B_1} \\
&\otimes\sum_{j=1}^{7}\left\|K_j|0\rangle\right\|^2|j\rangle_E\langle j|_E \\
&+\frac{1}{4}|\sigma_-\rangle_{A_1}\langle\sigma_-|_{A_1}\otimes|j_0\rangle_{A_2}\langle j_0|_{A_2}\otimes|\sigma_-\rangle_{B_1}\langle\sigma_-|_{B_1} \\
&\otimes\sum_{j=1}^{7}\left\|K_j|1\rangle\right\|^2|j\rangle_E\langle j|_E =
\end{aligned}
$$

$$
\begin{aligned}
&= \frac{1}{4}|\sigma_+\rangle_{A_1}\langle\sigma_+|_{A_1}\otimes|j_0\rangle_{A_2}\langle j_0|_{A_2}\otimes|\sigma_+\rangle_{B_1}\langle\sigma_+|_{B_1} \\
&\otimes\frac{1}{2}\left(p_2|5\rangle_E\langle 5|_E+p_3|6\rangle_E\langle 6|_E+(1-p_2-p_3)|7\rangle_E\langle 7|_E\right) \\
&+\frac{1}{4}|\sigma_+\rangle_{A_1}\langle\sigma_+|_{A_1}\otimes|j_{-2}\rangle_{A_2}\langle j_{-2}|_{A_2}\otimes|\sigma_+\rangle_{B_1}\langle\sigma_+|_{B_1} \\
&\otimes\frac{1}{2}\left(p_1|1\rangle_E\langle 1|_E+p_2|2\rangle_E\langle 2|_E+(1-p_1-p_2)|7\rangle_E\langle 7|_E\right) \\
&+\frac{1}{4}|\sigma_-\rangle_{A_1}\langle\sigma_-|_{A_1}\otimes|j_2\rangle_{A_2}\langle j_2|_{A_2}\otimes|\sigma_-\rangle_{B_1}\langle\sigma_-|_{B_1} \\
&\otimes\frac{1}{2}\left(p_1|1\rangle_E\langle 1|_E+p_2|2\rangle_E\langle 2|_E+(1-p_1-p_2)|7\rangle_E\langle 7|_E\right) \\
&+\frac{1}{4}|\sigma_-\rangle_{A_1}\langle\sigma_-|_{A_1}\otimes|j_0\rangle_{A_2}\langle j_0|_{A_2}\otimes|\sigma_-\rangle_{B_1}\langle\sigma_-|_{B_1} \\
&\otimes\frac{1}{2}\left(p_3|3\rangle_E\langle 3|_E+p_1|4\rangle_E\langle 4|_E+(1-p_1-p_3)|7\rangle_E\langle 7|_E\right)
\end{aligned}
\tag{S26}
$$

We define density operators $\rho^{X=0}_{A_1,A_2,B_1,E}$,$\rho^{X=1}_{A_1,A_2,B_1,E}$,$\rho^{X=2}_{A_1,A_2,B_1,E}$,$\rho^{X=3}_{A_1,A_2,B_1,E}$ derived from $\rho_{A_1A_2,B_1E}$ that depend on the encoding Alice choses (For example, $\rho^{X=0}_{A_1,A_2,B_1,E}$ derived from $\rho_{A_1A_2,B_1E}$ by collecting only terms satisfied $X=0$).

$$
\begin{aligned}
\rho^{X=0}_{A_1,A_2,B_1,E} &= \frac{1}{4}|\sigma_+\rangle_{A_1}\langle\sigma_+|_{A_1}\otimes|j_0\rangle_{A_2}\langle j_0|_{A_2}\otimes|\sigma_+\rangle_{B_1}\langle\sigma_+|_{B_1} \\
&\otimes\frac{1}{2}\left(p_2|5\rangle_E\langle 5|_E+p_3|6\rangle_E\langle 6|_E+(1-p_2-p_3)|7\rangle_E\langle 7|_E\right)
\end{aligned}
$$

$$\rho^{X=1}_{A_1,A_2,B_1,E} = \frac{1}{4}|\sigma_+\rangle_{A_1}\langle\sigma_+|_{A_1} \otimes |j_{-2}\rangle_{A_2}\langle j_{-2}|_{A_2} \otimes |\sigma_+\rangle_{B_1}\langle\sigma_+|_{B_1}$$
$$\otimes \frac{1}{2}(p_1|1\rangle_E\langle 1|_E + p_2|2\rangle_E\langle 2|_E + (1-p_1-p_2)|7\rangle_E\langle 7|_E)$$

$$\rho^{X=2}_{A_1,A_2,B_1,E} = \frac{1}{4}|\sigma_-\rangle_{A_1}\langle\sigma_-|_{A_1} \otimes |j_{2}\rangle_{A_2}\langle j_{2}|_{A_2} \otimes |\sigma_-\rangle_{B_1}\langle\sigma_-|_{B_1}$$
$$\otimes \frac{1}{2}(p_1|1\rangle_E\langle 1|_E + p_2|2\rangle_E\langle 2|_E + (1-p_1-p_2)|7\rangle_E\langle 7|_E)$$

$$\rho^{X=3}_{A_1,A_2,B_1,E} = \frac{1}{4}|\sigma_-\rangle_{A_1}\langle\sigma_-|_{A_1} \otimes |j_{0}\rangle_{A_2}\langle j_{0}|_{A_2} \otimes |\sigma_-\rangle_{B_1}\langle\sigma_-|_{B_1}$$
$$\otimes \frac{1}{2}(p_3|3\rangle_E\langle 3|_E + p_1|4\rangle_E\langle 4|_E + (1-p_1-p_3)|7\rangle_E\langle 7|_E)$$

Let's calculate the $I(X;E)_\rho$:

$$I(X;E)_\rho = H(E) - H(E|X) \tag{S27}$$

$$H(E|X) = \frac{1}{4}[H(E|X=0) + H(E|X=1) + H(E|X=2) + H(E|X=3)]$$

$$\begin{aligned} H(E) = &-\frac{p_2}{8}\log\frac{p_2}{8} - \frac{p_3}{8}\log\frac{p_3}{8} - \frac{(1-p_2-p_3)}{8}\log\frac{(1-p_2-p_3)}{8} - \frac{p_1}{8}\log\frac{p_1}{8} \\ &-\frac{p_2}{8}\log\frac{p_2}{8} - \frac{(1-p_1-p_2)}{8}\log\frac{(1-p_1-p_2)}{8} - \frac{p_1}{8}\log\frac{p_1}{8} - \frac{p_2}{8}\log\frac{p_2}{8} \\ &-\frac{(1-p_1-p_2)}{8}\log\frac{(1-p_1-p_2)}{8} - \frac{p_3}{8}\log\frac{p_3}{8} - \frac{p_1}{8}\log\frac{p_1}{8} \\ &-\frac{(1-p_1-p_3)}{8}\log\frac{(1-p_1-p_3)}{8} \end{aligned} \tag{S28}$$

$$H(E|X=0) = -\frac{p_2}{8}\log\frac{p_2}{8} - \frac{p_3}{8}\log\frac{p_3}{8} - \frac{(1-p_2-p_3)}{8}\log\frac{(1-p_2-p_3)}{8}$$

$$H(E|X=1) = -\frac{p_1}{8}\log\frac{p_1}{8} - \frac{p_2}{8}\log\frac{p_2}{8} - \frac{(1-p_1-p_2)}{8}\log\frac{(1-p_1-p_2)}{8}$$

$$H(E|X=2) = -\frac{p_1}{8}\log\frac{p_1}{8} - \frac{p_2}{8}\log\frac{p_2}{8} - \frac{(1-p_1-p_2)}{8}\log\frac{(1-p_1-p_2)}{8}$$

$$H(E|X=3) = -\frac{p_3}{8}\log\frac{p_3}{8} - \frac{p_1}{8}\log\frac{p_1}{8} - \frac{(1-p_1-p_3)}{8}\log\frac{(1-p_1-p_3)}{8}$$

Let's calculate the mutual information $I(X;Y)_\rho$:

Recall the density matrix $\rho_{A_1A_2,B_1,T}$:

$$\rho_{A_1,A_2,B_1,T} = \frac{1}{4}|\sigma_+\rangle_{A_1}\langle\sigma_+|_{A_1} \otimes |j_0\rangle_{A_2}\langle j_0|_{A_2} \otimes |\sigma_+\rangle_{B_1}\langle\sigma_+|_{B_1} \otimes |j_{-2}\rangle_T\langle j_{-2}|_T \qquad \text{"0"}$$

$$+\frac{1}{4}|\sigma_+\rangle_{A_1}\langle\sigma_+|_{A_1} \otimes |j_{-2}\rangle_{A_2}\langle j_{-2}|_{A_2} \otimes |\sigma_+\rangle_{B_1}\langle\sigma_+|_{B_1} \otimes |j_0\rangle_T\langle j_0|_T + \qquad \text{"1"} \tag{S29}$$

$$+\frac{1}{4}|\sigma_-\rangle_{A_1}\langle\sigma_-|_{A_1}\otimes|j_2\rangle_{A_2}\langle j_2|_{A_2}\otimes|\sigma_-\rangle_{B_1}\langle\sigma_-|_{B_1}\otimes|j_0\rangle_T\langle j_0|_T + \quad \text{"2"}$$

$$+\frac{1}{4}|\sigma_-\rangle_{A_1}\langle\sigma_-|_{A_1}\otimes|j_0\rangle_{A_2}\langle j_0|_{A_2}\otimes|\sigma_-\rangle_{B_1}\langle\sigma_-|_{B_1}\otimes|j_2\rangle_T\langle j_2|_T \quad \text{"3"}$$

For the beginning let's look at what Alice sent to Bob before passing through the channel, where we defined the OAM that is sent to Bob as $J_B = \{|j_0\rangle = |0\rangle, |j_2\rangle = |1\rangle, |j_{-2}\rangle = |2\rangle\}$.

If Bob measures $|0\rangle$:

$$\rho^{(J_B=0)}_{A_1,A_2,B_1} = \frac{1}{4}\left[\frac{1}{2}|\sigma_+\rangle_{A_1}\langle\sigma_+|_{A_1}\otimes|j_{-2}\rangle_{A_2}\langle j_{-2}|_{A_2}\otimes|\sigma_+\rangle_{B_1}\langle\sigma_+|_{B_1} + \frac{1}{2}|\sigma_-\rangle_{A_1}\langle\sigma_-|_{A_1}\otimes|j_2\rangle_{A_2}\langle j_2|_{A_2}\otimes|\sigma_-\rangle_{B_1}\langle\sigma_-|_{B_1}\right] \quad \text{(S30)}$$

If Bob measures $|1\rangle$:

$$\rho^{(J_B=1)}_{A_1,A_2,B_1} = \frac{1}{4}|\sigma_-\rangle_{A_1}\langle\sigma_-|_{A_1}\otimes|j_0\rangle_{A_2}\langle j_0|_{A_2}\otimes|\sigma_-\rangle_{B_1}\langle\sigma_-|_{B_1} \quad \text{(S31)}$$

If Bob measures $|2\rangle$:

$$\rho^{(J_B=2)}_{A_1,A_2,B_1} = \frac{1}{4}|\sigma_+\rangle_{A_1}\langle\sigma_+|_{A_1}\otimes|j_0\rangle_{A_2}\langle j_0|_{A_2}\otimes|\sigma_+\rangle_{B_1}\langle\sigma_+|_{B_1} \quad \text{(S32)}$$

We defined the channel as before:

$$\rho_{A_1,A_2,B_1,B_2} = (\mathbb{1}\otimes\mathcal{N}_{T\to B_2})\rho_{A_1,A_2,B_1,T} = \sum_{j=1}^{7}(\mathbb{1}\otimes K_j)\rho_{A_1,A_2,B_1,T}(\mathbb{1}\otimes K_j)^\dagger =$$

$$= \frac{1}{4}|\sigma_+\rangle_{A_1}\langle\sigma_+|_{A_1}\otimes|j_0\rangle_{A_2}\langle j_0|_{A_2}\otimes|\sigma_+\rangle_{B_1}\langle\sigma_+|_{B_1} \otimes\frac{1}{2}\left(p_2|0\rangle_{B_2}\langle 0|_{B_2} + p_3|1\rangle_{B_2}\langle 1|_{B_2} + (1-p_2-p_3)|2\rangle_{B_2}\langle 2|_{B_2}\right)$$

$$+\frac{1}{4}|\sigma_+\rangle_{A_1}\langle\sigma_+|_{A_1}\otimes|j_{-2}\rangle_{A_2}\langle j_{-2}|_{A_2}\otimes|\sigma_+\rangle_{B_1}\langle\sigma_+|_{B_1} \otimes\frac{1}{2}\left(p_1|1\rangle_{B_2}\langle 1|_{B_2} + p_2|2\rangle_{B_2}\langle 2|_{B_2} + (1-p_1-p_2)|0\rangle_{B_2}\langle 0|_{B_2}\right)$$

$$+\frac{1}{4}|\sigma_-\rangle_{A_1}\langle\sigma_-|_{A_1}\otimes|j_2\rangle_{A_2}\langle j_2|_{A_2}\otimes|\sigma_-\rangle_{B_1}\langle\sigma_-|_{B_1} \otimes\frac{1}{2}\left(p_1|1\rangle_{B_2}\langle 1|_{B_2} + p_2|2\rangle_{B_2}\langle 2|_{B_2} + (1-p_1-p_2)|0\rangle_{B_2}\langle 0|_{B_2}\right)$$

$$+\frac{1}{4}|\sigma_-\rangle_{A_1}\langle\sigma_-|_{A_1}\otimes|j_0\rangle_{A_2}\langle j_0|_{A_2}\otimes|\sigma_-\rangle_{B_1}\langle\sigma_-|_{B_1} \otimes\frac{1}{2}\left(p_3|2\rangle_{B_2}\langle 2|_{B_2} + p_1|0\rangle_{B_2}\langle 0|_{B_2} + (1-p_1-p_3)|1\rangle_{B_2}\langle 1|_{B_2}\right)$$

$$\text{(S33)}$$

Let's define new state $|e\rangle$ which represent the loss after the channel. For the mutual information $I(X;Y)_\rho = H(Y) - H(Y|X)$. The probability that Bob gets the logic number $\{0,1,2,3,e\}$ derived by collecting the probability for each logic number.

$$P_{|0\rangle} = \frac{p_2}{8} + \frac{1 - p_2 - p_3}{8} = \frac{1 - p_3}{8}$$

$$P_{|1\rangle} = \frac{1 - p_1 - p_2}{8} + \frac{p_2}{8} = \frac{1 - p_1}{8}$$

$$P_{|2\rangle} = \frac{p_1}{8} + \frac{1 - p_1 - p_2}{8} = \frac{1 - p_2}{8}$$

$$P_{|3\rangle} = \frac{p_1}{8} + \frac{1 - p_1 - p_3}{8} = \frac{1 - p_3}{8}$$

$$P_{|e\rangle} = \frac{p_2}{8} + \frac{p_3}{8} + \frac{p_2}{8} + \frac{p_1}{8} = \frac{2p_2 + p_1 + p_3}{8}$$

$$H(Y) = -\left(\frac{1 - p_3}{8}\right)\log\left(\frac{1 - p_3}{8}\right) - \left(\frac{1 - p_1}{8}\right)\log\left(\frac{1 - p_1}{8}\right) - \left(\frac{1 - p_3}{8}\right)\log\left(\frac{1 - p_3}{8}\right)$$
$$- \left(\frac{2p_2 + p_1 + p_3}{8}\right)\log\left(\frac{2p_2 + p_1 + p_3}{8}\right)$$

$$H(Y|X) = \frac{1}{3}[H(Y|X = 0) + H(Y|X = 1) + H(Y|X = 2) + H(Y|X = 3)] \quad \text{(S34)}$$

From the density matrices the probabilities are:

$$p(Y = 0|X = 0) = \frac{1 - p_2 - p_3}{8}$$
$$p(Y = 1|X = 0) = \frac{p_2}{8}$$
$$p(Y = 2|X = 0) = 0$$
$$p(Y = 3|X = 0) = 0$$
$$p(Y = e|X = 0) = \frac{p_3}{8}$$

$$p(Y = 0|X = 1) = \frac{p_2}{8}$$
$$p(Y = 1|X = 1) = \frac{1 - p_1 - p_2}{8}$$
$$p(Y = 2|X = 1) = 0$$
$$p(Y = 3|X = 1) = 0$$
$$p(Y = e|X = 1) = \frac{p_1}{8}$$

$$p(Y = 0|X = 2) = 0$$
$$p(Y = 1|X = 2) = 0$$
$$p(Y = 2|X = 2) = \frac{1 - p_1 - p_2}{8}$$
$$p(Y = 3|X = 2) = \frac{p_1}{8}$$
$$p(Y = e|X = 2) = \frac{p_2}{8}$$

$$p(Y = 0|X = 3) = 0$$
$$p(Y = 1|X = 3) = 0$$

$$p(Y=2|X=3)=\frac{p_1}{8}$$

$$p(Y=3|X=3)=\frac{1-p_1-p_3}{8}$$

$$p(Y=e|X=3)=\frac{p_3}{8}$$

The entropies:

$$H(Y|X=0)=-\left(\frac{1-p_2-p_3}{8}\right)\log\left(\frac{1-p_2-p_3}{8}\right)-\left(\frac{p_2}{8}\right)\log\left(\frac{p_2}{8}\right)-\left(\frac{p_3}{8}\right)\log\left(\frac{p_3}{8}\right)$$

$$H(Y|X=1)=-\left(\frac{p_2}{8}\right)\log\left(\frac{p_2}{8}\right)-\left(\frac{p_1}{8}\right)\log\left(\frac{p_1}{8}\right)-\left(\frac{1-p_1-p_2}{8}\right)\log\left(\frac{1-p_1-p_2}{8}\right)$$

$$H(Y|X=2)=-\left(\frac{1-p_1-p_2}{8}\right)\log\left(\frac{1-p_1-p_2}{8}\right)-\left(\frac{p_1}{8}\right)\log\left(\frac{p_1}{8}\right)-\left(\frac{p_2}{8}\right)\log\left(\frac{p_2}{8}\right)$$

$$H(Y|X=3)=-\left(\frac{p_1}{8}\right)\log\left(\frac{p_1}{8}\right)-\left(\frac{1-p_1-p_3}{8}\right)\log\left(\frac{1-p_1-p_3}{8}\right)-\left(\frac{p_3}{8}\right)\log\left(\frac{p_3}{8}\right)$$

We use all the terms and calculate the Key Rate for the new channel.

We compute the key rate for the noisy channels we analyze – the erasure channel (Fig. S3(a)) and for the newly introduced physical channel (Fig. S3(b)). Fig. S3(b) illustrates the key rate for the three probabilities, with colors indicating the calculated key rate values.

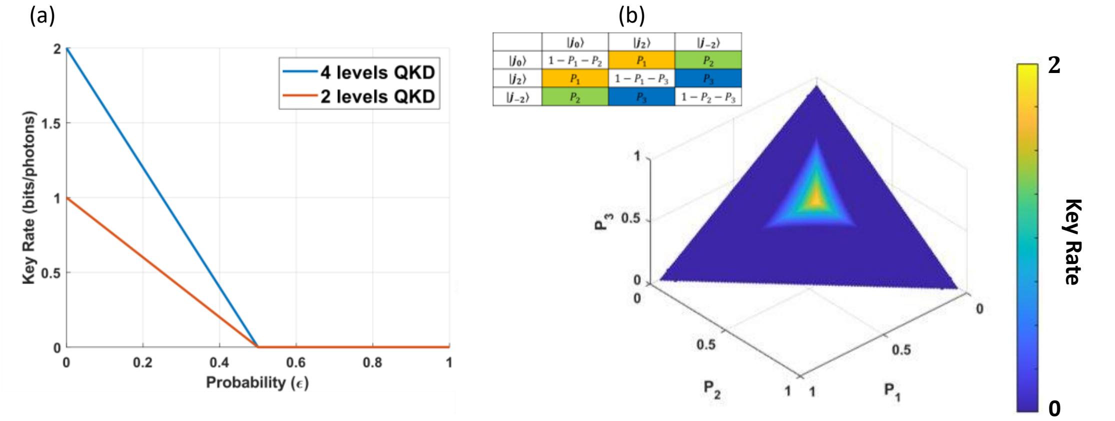

Figure S3: The protocol key rate analysis (a) key rate for eraser channel, compared with the standards BB84 protocol with two digits, and the enhanced key rate with 4 digits. (b) key rate for our new noisy channel we formulated as function of the probabilities a 3D plot.

Approximate Mutual Unbiasedness of Polarization–OAM Bases

In our protocol the two families of states (generated with circularly and linearly polarized pumps) define two distinct measurement bases for the polarization-OAM Hilbert space. Approximate mutual unbiasedness is desirable because it ensures nearly uniform outcome statistics and maximal complementarity. Small deviations from perfect unbiasedness do not invalidate a QKD protocol but affect the expected error rates and, consequently, the extractable key rate.

The circular-polarization basis used in our formalism is formed by product states of circular polarization and orthonormal OAM modes, while the linear-polarization basis produced by a linearly polarization corresponds to Hadamard mixing of the polarization DoF together with equal-weight superpositions of the orthonormal OAM modes (the TAM-preserving combinations described in the text). For a 4-dimensional states (i.e., the qudits encoded in the photons emitted from the nonlinear interaction), the

overlap between any circular-basis vector and any linear-basis vector equals $\frac{1}{2}$ and $\frac{1}{4}$ amplitude probability. Let us show this explicitly:

The polarization circular basis: $|\sigma_+\rangle$, $|\sigma_-\rangle$. The linear basis: $|H\rangle = \frac{1}{\sqrt{2}}(|\sigma_+\rangle + |\sigma_-\rangle)$, $|V\rangle = \frac{1}{\sqrt{2}}(|\sigma_+\rangle - |\sigma_-\rangle)$. From the 2 SPP modes we get (that is, J_1 and J_{-1}) we are getting 4 non-overlapping OAM modes in the emitted photons. These OAM states are orthonormal, $\langle j_m|j_k\rangle = 0$. The set of our 4 orthogonal states is thus: $\{|\sigma_+, j_0\rangle, |\sigma_+, j_{-2}\rangle, |\sigma_-, j_0\rangle, |\sigma_-, j_2\rangle\}$. Similarly, for the linear-polarization basis, we are getting equal-wight OAM superpositions associated with H and V. That is: $\{|H, HB_{20}\rangle, |V, HB_{11}\rangle, |H, HB_{11}\rangle, |V, HB_{02}\rangle\} = \{|H, (-j_2 + 2j_0 + j_{-2})\rangle, |V, (j_2 + j_{-2})\rangle, |H, (j_2 + j_{-2})\rangle, |V, (-j_2 + 2j_0 + j_{-2})\rangle\}$. Then, for the overlap calculation, the inner product gives us:

$$\langle|\sigma_+, j_0\rangle|H, (-j_2 + 2j_0 + j_{-2})\rangle = \frac{1}{\sqrt{2}} \cdot \sqrt{\frac{2}{3}} = \frac{1}{\sqrt{3}} \approx 0.57$$

For the products with HB_{20}, and $\frac{1}{2}$ for the products with HB_{11}. Thus, the probability is $|\langle|\sigma_+, j_0\rangle|H, (-j_2 + 2j_0 + j_{-2})\rangle|^2 = \frac{1}{3}$ or $|\langle|\sigma_+, j_0\rangle|H, (j_2 + j_{-2})\rangle|^2 = \frac{1}{4}$

Although the overlaps are not all exactly equal (as in an ideal mutually unbiased basis), the values we obtain ($1/3$ and $1/4$) are close to the ideal $1/4$. Thus, the two bases can be regarded as approximately mutually unbiased, and can generate a secure key-rate. For QKD purposes, this level of complementarity is sufficient to guarantee nearly uniform outcome statistics when measuring in the non-matching basis, with the small deviations entering as additional noise/error terms in the key-rate analysis.

Section D: Photon-Pair Emission Probability in the Air/SiO₂/Si/Au Hybrid

We consider a planar stack (top to bottom) composed of air / Si (thickness t_{Si} = 60 (nm)) / SiO2 (thickness t_{ox} = 2 (nm)) / Au (thickness t_{Au}=220 (nm)), effectively semi-infinite. A circular grating with N_g periods supply in-plane momentum. A classical, pulsed pump at wavelength λ_p = 1550 (nm) temporally overlaps with a single-quantum hybrid plasmon–photon polariton (HPP) at λ_{hpp} = 808 (nm). Through a third-order process, one pump photon and one HPP quantum are annihilated, resulting in the creation of two free-space photons, as described by the Hamiltonian eq. 2 in the main text.

For the hybrid mode, the Si contribution dominates due to confinement in the 60 nm Si layer and the strong coupling through the 2 nm spacer. We Assume an instantaneous, scalar $\chi^{(3)}(r)$. the interaction Hamiltonian reads: $H_{int}(t) = \hbar \sum_{\{\mu_1,\mu_2\}} G_{\{\mu_1\mu_2\}}(t) \cdot \hat{a}^\dagger_{\{\mu_1\}}\, \hat{a}^\dagger_{\{\mu_2\}\hat{b}} \cdot exp[i\Delta_{\{\mu_1\mu_2\}}t] + h.c.$ Detuning: $\Delta_{\{\mu_1\mu_2\}} = \omega_{\{\mu_1\}} + \omega_{\{\mu_2\}} - (\omega_p + \omega_{HPP})$

The coupling can be separated into temporal and spatial components:

$$G_{\{\mu_1\mu_2\}}(t) = \left(\frac{3}{4}\right) \cdot \left[\frac{\mathcal{E}_p(t)}{2\varepsilon_0\hbar}\right] \cdot \sqrt{\omega_{\{\mu_1\}}\omega_{\{\mu_2\}}\omega_{HPP}} \cdot M_{\{\mu_1\mu_2\}}$$

where the spatial overlap is

$$M_{\{\mu_1\mu_2\}} = \int \chi^3(r) \cdot (u_{\{\mu_1\}} \cdot u_{\{\mu_2\}}) \cdot (u_{HPP} \cdot u_p) \cdot exp[i\Delta k \cdot r]dV$$

with $\Delta k = k_{\{\mu_1\}} + k_{\{\mu_2\}} - k_p - k_{HPP}$.

Initial state: one HPP quantum, radiation vacuum, classical pump.

$$|\Psi\rangle \approx |0\rangle_{rad}|1\rangle_{HPP} - \left(\frac{i}{\hbar}\right) \int H_{int}(t)dt \cdot |0\rangle_{rad}|1\rangle_{HPP} =$$

$$= |0\rangle|1_{HPP}\rangle + \sum_{\{\mu_1\mu_2\}} \xi_{\{\mu_1\mu_2\}} \left|1_{\{\mu_1\}}, 1_{\{\mu_2\}}\right\rangle |0_{HPP}\rangle$$

The Joint spectral amplitude is $\xi_{\{\mu_1\mu_2\}} = -i \int G_{\{\mu_1\mu_2\}}(t) \cdot \mathrm{e}^{i\,\Delta_{\{\mu_1\mu_2\}}t} dt$. Let the HPP temporal mode be $f_{HPP}(t)$ with lifetime γ_{HPP}^{-1}. The effective time envelope is $\mathcal{E}_p(t) f_{HPP}(t)$. In frequency: $\xi(\omega_1, \omega_2) \propto \left[\tilde{\mathcal{E}}_p \star \tilde{f}_{HPP}\right](\omega_1, +\omega_2) \times \Phi(\omega_1, \omega_2)$, where Φ includes the longitudinal sinc, $\Gamma_\perp$, polarizations, and grating factors. Degenerate emission: $\omega_1 = \omega_2 = \frac{(\omega_p + \omega_{HPP})}{2}$. For λ_p=1550 (nm) and λ_{HPP}= 808 (nm), the degenerate wavelength is ≈ 1060 (nm).

Pair probability into a collection manifold C, per single HPP quantum that overlaps the pump: $P_{pair} = \sum_{\{(\mu_1\mu_2) \in C\}} \left|\xi_{\{\mu_1\mu_2\}}\right|^2$. Define effective temporal overlap $\tau_{eff} = [\int \frac{\mathcal{E}_p(t) f_{HPP}(t)}{\mathcal{E}_{p,eff}} \mathrm{d}t$.

Experiment-ready scaling:

$$P_{pair} \approx \frac{9}{64} N_{HPP} \frac{\omega_1 \omega_2 \omega_{HPP}}{\varepsilon \hbar^2} \left(\mathcal{E}_{p,eff} \tau_{eff}\right)^2 \cdot \left|M_{eff}\right|^2 \cdot D \cdot {\eta_{out}}^2$$

Where:

• N_{HPP} = mean HPP quanta per overlap event (N_{HPP}=1 for a single quantum). With an injected flux F_{HPP} (single HPPs s^{-1}), the pair rate is $R_{pair} = F_{HPP} \cdot P_{pair}$.

• $\mathcal{E}_{p,eff}^2 = \frac{2\mathrm{I_{peak}}}{(\mathrm{n_{eff}} \cdot \mathrm{c} \cdot \varepsilon_0)}$ Pump scaling is linear in intensity (one classical pump).

• The dimensionless modal overlap is: $\left|M_{eff}\right|^2 = \left[\frac{\Gamma_\perp^2}{(\mathrm{v_g}\,\mathrm{A_{eff}})^2}\right] = \left|\sum_\ell \chi_\ell^3 \alpha_\ell\right|^2 \cdot \left[\mathrm{L} \cdot \mathrm{sinc}\left(\Delta \mathrm{k}_\parallel \frac{\mathrm{L}}{2}\right)\right]^2$, with A_{eff} an effective transverse area and α_ℓ pump-mode overlap in each layer.

• D = collection mode-density (bandwidth × solid angle).

• η_{out} = single-photon outcoupling/collection efficiency (squared for pairs).

We assume synchronized pump and HPP pulses, with pulse rate f_{rep} and small HPP flux $\left(F_{HPP} \ll f_{rep}\right)$. The generation rate is therefore $R_{pair} = F_{HPP} P_{pair}$.

Using representative system parameters:

- Laser: 140 (fs) (FWHM) Gaussian pump, $f_{rep} = 80\ (MHz), P_{avg} = 300\ (mW)$
- Pulse energy $E_{pulse} = \frac{P_{avg}}{f_{rep}} = 3.75 \cdot 10^{-9}\ (J)$, Gaussian peak power: $P_{peak} \approx 2.52 \cdot 10^4\ (W)$.
- Volume of the nonlinear interaction: $V = 5 \cdot 10^{-19}\ (m^3)$.
- Interaction length uniform along the Si layer $L_{hybrid} \sim 60$ (nm).

- Interaction area $A = 10 \cdot \pi^2\ 10^{-6}\ (m^2)$.
- $\chi_{si}^3 \approx 5 \cdot 10^{-18}\ (m^2\ V^{-2}\ )$.
- Nonlinear parameter $\gamma = \frac{2\pi n_{si}}{\lambda_{pump}A} \approx 1.23 \frac{1}{Wm}$
- $G = \left(\mathcal{E}_{p,eff}\tau_{eff}\right)^2 \cdot \left|M_{eff}\right|^2 = 3.44 \cdot 10^{-3}$

For a realistic HPP flux the number of events: $N_{event} \approx F_{HPP} = 1 \cdot 10^7\ (s^{-1})$

$$R_{pair}^{hybrid} = N_{event}p_{pair} = 1 \cdot 10^7\ \cdot (3.44 \cdot 10^{-3})^2 \approx 118\ (s^{-1})$$

Section E: Losses and background processes

Plasmonic systems are inherently lossy, and additional background processes such as multiphoton photoluminescence and Raman-like scattering can, in principle, reduce the fidelity of the generated quantum state. In this section we elaborate on the losses and background processes that can affect our setup:

Surface plasmons suffer from absorption and radiative losses, which reduce photon survival probability. Background photons arise, for example, from multiphoton photoluminescence: when multiple photons are absorbed simultaneously, electrons are excited to higher energy states and subsequently relax, emitting broadband, incoherent photons that act as noise. If detectors cannot distinguish these counts from the true signal, the observed density matrix becomes a mixture of the ideal state and noise. Narrowband spectral filters can mitigate these effects by isolating the desired signal wavelength. The resulting uncorrelated background photons increase accidental coincidences and reduce visibility in correlation measurements.

Specifically, for our scheme: SPPs on metals exhibit large ohmic loss. Only a fraction $\eta_{rad} = \Gamma_{rad}/\Gamma_{tot}$ of the SPP energy radiates into the desired far-field modes; the remainder is lost to heat. This reduces the efficiency and introduces mode-dependent amplitude bias if η_{rad} varies with OAM order. Loss therefore directly impacts fidelity, acting as an amplitude damping channel: for a pure biphoton (polarization-OAM) state $|\psi\rangle$, mode-dependent loss converts the ideal density matrix ρ_{ideal} into a mixture ρ_{obs} where components with larger loss are attenuated, decreasing purity and the overlap $F = \langle\psi|\rho_{obs}\ |\psi\rangle$. To mitigate these effects in practice, for experimental demonstration, it is important to use narrowband spectral filters to isolate the signal wavelength, and spatial or mode filtering via mode sorters and single-mode fibers to select only the desired OAM channels. Moreover, to overcome the background noise, high efficiency and low dark-count SNSPDs are necessary. Together, these measures suppress both amplitude damping and uncorrelated background contributions to a detectable level.

References

1. R. Renner, "Security of Quantum Key Distribution," Thesis (2005).

2. J. Wang, S. Chen, and J. Liu, "Orbital angular momentum communications based on standard multi-mode fiber (invited paper)," APL Photonics **6**, (2021).
